# Isolating the role of researcher observation on reactivity to the measurement of physical activity

**DOI:** 10.1111/aphw.12630

**Published:** 2024-12-19

**Authors:** Laura M. König, Kristen Pasko, Kiri Baga, Raj Harsora, Danielle Arigo

**Affiliations:** ^1^ Faculty of Psychology University of Vienna Austria; ^2^ Faculty of Life Sciences: Food, Nutrition and Health University of Bayreuth Germany; ^3^ Department of Psychology Rowan University New Jersey USA

**Keywords:** measurement reactivity, physical activity, researcher observation, social comparison orientation, wearables

## Abstract

Reactivity to physical activity (PA) measurement may result from the introduction of a measurement device, researcher observation, or both. Accessing data from prior to study enrollment afforded the rare opportunity to compare behavior during versus *prior to* participation. This study introduced researcher observation among adults who owned their own PA monitoring device, to test whether measurement reactivity can also be observed in experienced PA trackers, by comparing their data from before versus after the introduction of observation. In addition, the salience of researcher observation was manipulated to test for potential effects. Participants were 252 adults in the U.S. They completed two electronic surveys 14 days apart, in which they recorded steps per day as collected by their PA monitors over the previous 14 days. At the end of the first survey, they were randomized to view messages, which differed in emphasis on repeating entry of step data (i.e., “low” vs. “high” salience of researcher observation). Daily step counts did not change between 14‐day reporting periods, though patterns differed by gender and starting level of PA. Patterns did not differ between experimental conditions. Overall, introducing researcher observation without introducing an unfamiliar measurement device results in no meaningful reactivity with respect to PA.

## INTRODUCTION

Considerable evidence documents the existence of research participation effects, or changes in people's emotions, cognitions, and behavior due to taking part in a research study (McCambridge et al., 2014). This is thought to occur because participants' awareness of the experience of interest increases during a research study, which induces self‐regulatory mechanisms that lead to an adjustment of the behavior toward what is assumed to be desired or healthy (Moos, 2008). These effects are often attributed to *measurement reactivity*, or changes in the experience of interest due to the introduction of assessment protocols (and often, unfamiliar measurement devices or procedures; French & Sutton, 2010; French et al., 2021).

Yet, reactivity may occur in response to the mere awareness that “someone else is watching” and will see the data a participant provides ‐ namely, the researchers (e.g., Hawthorne effect; see Christiansen et al., 2023; French et al., 2021). This can occur even when researchers do not explicitly highlight their intention to review particular aspects of participant responses or their role in reviewing participant data; it may be a mere and natural consequence of joining a research study (McCambridge et al., 2014). In most studies, however, it is impossible to separate the contributions of response to measurement from response to researcher observation. This complicates drawing conclusions about the underlying psychological mechanisms of measurement reactivity as well as about means to mitigate its influence. Specifically, it is not clear what effect just joining the study and knowing what to expect can impact a behavior of interest, relative to more explicit emphasis on researcher observation of behavioral data. Thus, researchers may heighten observation effects inadvertently, and improving our understanding of even subtle adjustments to study instructions would be useful for informing future research.

With respect to the measurement of physical activity (PA) behavior, PA often increases and/or sedentary time decreases during study participation; participation is thought to activate self‐presentation and social desirability biases to be more in line with widely known health guidelines (Barta et al., 2012; French et al., 2021). Here, reactivity can occur in response to the novelty of wearing a device that tracks PA parameters (e.g., wristwatch that collects steps per day; Arigo & König, 2024) and provides self‐monitoring data in real‐time. PA reactivity may also result from knowledge of researcher observation in the broader context of research participation.

Available evidence indicates that measurement reactivity effects are typically small and attenuate after the first one or two days of study participation (König et al., 2022). This is true even when PA and its potential determinants are assessed repeatedly over short intervals (e.g., hours), as in studies that use ecological momentary assessment and other experience sampling designs (Arigo & König, 2024; Maher et al., 2024). Further, these effects tend to be present only for aspects of PA that capture overall movement, such as daily step counts and time spent in sedentary versus non‐sedentary activities (König et al., 2022). This is likely because overall movement is much easier to modify than minutes of activity at particular intensities and modifications can occur without conscious intention (which is rarely the case for minutes of light, moderate, or vigorous PA; Cheval & Boisgontier, 2021). This is thought to resolve quickly as participants' awareness shifts away from the measurement process as they habituate to it (Barta et al., 2012), and it is assumed ‐ though rarely empirically demonstrated ‐ that behavior returns to participants' typical levels later in the observation period. As it is common for researchers to average across days of observation to determine a participant's “typical” PA, small changes at the start of observation periods may skew averages and lead to imprecise (if not inaccurate) conclusions.

For this reason, some researchers recommend drastic actions such as adding initial days of observation that will be dropped from statistical analyses or concealing the true purpose of a measurement device (e.g., Clemes & Parker, 2009; see French & Sutton, 2010). In contrast, researchers have repeatedly hypothesized that familiarity with a PA monitoring device may mitigate PA measurement reactivity (Arigo & König, 2024; König et al., 2022). As participants are already familiar with the device and with self‐monitoring feedback, continued use of the same device should not induce a shift in awareness that causes subsequent changes in behavior. This lack of change would suggest that reactivity would indeed rather be a reaction to the introduction of *measurement*. However, if behavior still changed among participants who used a device prior to enrolling in the study, this would point toward reactivity as a reaction to the study *context* (e.g., knowledge of being observed by the researchers; see Arigo & König, 2024). Understanding the role of researcher observation, as well as the extent to which participants respond to subtle differences in how researchers present the study, could point to optimal (and less drastic) measures to mitigate any potential reactivity.

Finally, a range of individual characteristics have been hypothesized to exacerbate measurement reactivity. König et al. (2022) identified four studies that tested the potential moderating effects of gender on reactivity to measuring steps that found no gender differences, but another study focusing on activity counts, which are highly correlated with steps (Sartini et al., 2015), reported reactivity in girls, but not boys (Ho et al., 2013). In general, women seem to be more prone to social desirability bias when reporting on health behaviors (Bernardi & Guptill, 2008; Hebert et al., 1997) and may be more likely than men to (initially) adjust their behavior in the context of research participation. Similarly, those who are more interested in evaluating their status relative to peers (i.e., higher social comparison orientation [SCO]; Gibbons & Buunk, 1999) might also be more interested in managing others' impressions of their relative status than those who are less interested in comparative evaluation. Pre‐participation levels of the behavior of interest might also be relevant: individuals who perform poorly relative to relevant guidelines or (perceived) expectations have more room to improve on the target behavior than those who perform well. Thus, women (vs. men), those with stronger (vs. weaker) SCO, and those with lower (vs. higher) starting levels of PA might be more motivated or feel more pressure to change their behavior in the context of research participation (Barta et al., 2012).

## AIMS OF THE PRESENT STUDY

As an initial step toward determining the unique role of researcher observation in PA research participation effects and its response to manipulation, this preregistered two‐part study was designed to test for reactivity to study enrollment (and thus, researcher observation) among adults who already use their own personal PA monitors. Using existing data from PA monitors provides a unique opportunity to study measurement reactivity: it allows for the separation of reactivity to a measurement device from reactivity to researcher observation, and it enables comparison of behavior during participation to *prior* behavior (which is difficult to access). It also removes the potential effect of intensive assessment of PA predictors; in experience sampling studies, these are typically assessed with self‐report questions multiple times per day (Dunton, 2017). Although PA may be assessed continuously in these studies with a commercial device (which promotes habituation to measurement), answering questions about PA every few hours may provide reminders that increase the salience of PA (and uniquely contribute to reactivity). Thus, the present study introduced only observation from the research team, and the salience of this observation was experimentally manipulated by assigning participants to a “low” or “high” salience condition at one point in time (i.e., on the day participants completed the initial study activity).

To do this, we collected PA behavior as logged by a personal PA monitor after entry into the study (Days 15–28), compared to the two weeks prior to study participation (Days 1–14). Specifically, we tested whether:

1. Average step counts were higher during study participation (Days 15–28) vs. before (Days 1–14),
2. Step counts were higher on day 1 (Day 15) vs. before and on the following days in the study (Days 1–14 and 17–28),
3. Step counts were higher on days 1 and 2 of study participation (Days 15–16) vs. before and on the following days in the study (Days 1–14 and 17–28), and
4. Patterns of step counts differ depending on the salience of researcher observation (low vs. high via random assignment, introduced on the first day of study participation [Day 15]).

The analyses above were co‐registered on the Open Science Framework (https://osf.io/s2rby). Follow‐up exploratory aims were to test for reactivity patterns among only those who completed the study as intended and for the moderating effects of gender, average PA during the pre‐study period (Days 1–14), and social comparison orientation on these patterns.

## METHODS

### Recruitment and participants

For this initial stage of work, we selected a target sample size of 250 participants based on balancing feasibility and resource availability with achieving adequate power for the statistical analyses (described below; Murayama et al., 2022). People were eligible for the study if they were at least 18 years old, had a personal PA monitoring device (i.e., wearable monitor, smartwatch, or smartphone app), and had been recording their daily step count for at least two weeks before enrollment in the study. Recruitment targeted two populations who use PA monitors. The first was undergraduate students taking introductory psychology courses at a large public university in the northeastern U.S. The study was advertised to all students in this course and students received course credit for participation. The second was the wider population of adults, who were recruited via social media, professional listservs, and announcements at the supporting institution (for students not enrolled in introductory psychology courses, faculty, and staff).^1^ These participants were entered into a drawing for $50 at the Amazon store.

Of the 436 individuals who completed Phase 1 and met the criteria for inclusion (see below), 252 completed Phase 2 and were included in analyses (*M*_*Age*_ = 30.47, *M*_*BMI*_ = 25.83 kg/m^2^). The majority of participants identified as women (71%), white (78%), using a wristband PA monitor (72%), and using this device for more than one year (69%). Average steps per day for the pre‐study period ranged from 878 to 19,133 (*M* = 8,061, *SD* = 3,328). A total of 130 participants were assigned to the low‐salience condition and 122 individuals were assigned to the high‐salience condition. Table 1 shows demographic information for the full sample; there were no significant differences in demographics between experimental conditions (*p*s ≥ .093). One hundred and ten participants completed the second survey on Day 15, as intended (44%); five participants completed before Day 15 (2%), and the remaining participants completed on Days 16 or 17 (137; 54%).

**TABLE 1:** Sociodemographic characteristics of the full sample and by condition.

	Response option	Full sample (*N* = 252)	Low saliency condition (*n* = 130)	High saliency condition (*n* = 122)	Comparison of conditions
Age (M, SD)		30.47, 12.43	30.58, 12.39	30.36, 12.52	*t*(242) = 0.14, *p* = .889
Gender (%)	Women	71.0%	66.2%	76.2%	Χ^2^(*df* = 4) = 8.00, *p* = .092
	Men	26.2%	28.5%	23.8%	
	Gender neutral	0.4%	0.8%	0.0%	
	Non‐binary	1.6%	3.1%	0.0%	
	Would rather not say	0.8%	1.5%	0.0%	
Ethnic or racial heritage (%)	American Indian or Alaska native	0.4%	0.8%	0.0%	Χ^2^(*df* = 5) = 3.21, *p* = .668
	East Asian	2.4%	3.1%	1.7%	
	Black or African American	8.4%	10.0%	6.6%	
	White	77.7%	76.2%	79.3%	
	Multiracial	4.4%	4.6%	4.1%	
	Other	6.8%	5.4%	8.1%	
Ethnicity (%)	Not Hispanic or Latinx	88.8%	91.5%	86.1%	Χ^2^(*df* = 1) = 1.85, *p* = .174
	Hispanic or Latinx	11.2%	8.5%	13.9%	
BMI (M, SD)		25.83, 6.11	25.81, 5.63	25.85, 6.62	*t*(242) = −0.06, *p* = .995
Physical activity tracking device (%)	Watch/wrist band	71.8%	70.8%	73.0%	Χ^2^(*df* = 5) = 2.01, *p* = .848
	Ring/smart jewelry	0.8%	0.8%	0.8%	
	Smart clothing	0.4%	0.8%	0.0%	
	Pedometer	0.4%	0.8%	0.0%	
	Phone application	23.8%	23.8%	23.8%	
	Other	2.8%	3.1%	2.5%	
Tracker use duration (%)	1 month or less	7.2%	7.0%	7.4%	Χ^2^(*df* = 5) = 8.43, *p* = .134
	2–3 months	5.6%	8.5%	2.5%	
	4–5 months	4.8%	4.7%	4.9%	
	6 months	4.0%	1.6%	6.6%	
	6 months – 1 year	9.6%	8.5%	10.7%	
	More than 1 year	68.9%	69.8%	69.0%	
Use consistency (M, SD)		3,59, 0.97	5.35, 1.00	5.43, 0.94	*t*(250) = −0.59, *p* = .995
Average steps for pre‐study period, reported in Phase 1		8060.81, 3328.06	8008.81, 3462.75	8115.79, 3192.81	*t*(249) = −0.25, *p* = .800
Median of steps for pre‐study period, reported in Phase 1		7514.79	7266.14	7654.65	
Physical activity levels typical in past 14 days, reported in Phase 1	No	17.5%	16.2%	18.9%	Χ^2^(*df* = 1) = 0.32, *p* = .573
	Yes	82.5%	83.8%	81.1%	
Physical activity levels typical in past 14 days, reported in Phase 2	No	22.5%	24.5%	19.7%	Χ^2^(*df* = 1) = 1.17, *p* = .279
	Yes	77.5%	74.6%	80.3%	
Covid‐19 reported in Phase 1	No	99.2%	99.2%	99.2%	Χ^2^(*df* = 1) = 0.00, *p* = .964
	Yes	0.8%	0.8%	0.8%	
Covid‐19, reported in Phase 2^1^	No	100.0%	100.0%	100.0%	‐
	Yes	0.0%	0.0%	0.0%	
Other respiratory illness, reported in Phase 1	No	93.3%	93.1%	93.4%	Χ^2^(*df* = 1) = 0.01, *p* = .908
	Yes	6.7%	6.9%	6.6%	
Other respiratory illness, reported in Phase 2	No	90.9%	90.0%	91.7%	Χ^2^(*df* = 1) = 0.23, *p* = .634
	Yes	9.1%	10.1%	8.3%	

*Note*: ^1^ Since no incidents were reported in Phase 2, group comparisons were not possible.

### Design and procedure

Data for this between‐subjects experimental two‐part study were collected between October 2021 and June 2024. All procedures were approved by the supporting university's Institutional Review Board. Participants provided informed consent by ticking a box on the first page of the first online survey. Data analysis procedures were co‐registered in August 2022 prior to the inspection of the data by the analyst (https://osf.io/s2rby). Participants used personal PA tracking devices (i.e., wearable devices or smartphone applications) to capture activity step counts. Commercial devices typically allow users to review their step counts for several weeks to months, often up until the device was first used. Interested individuals were directed to an initial survey (Phase 1), in which they entered their activity step counts as reported by their PA monitor for the prior 14 days (Days 1–14, occurred prior to enrollment). Two weeks later, participants received a second questionnaire and again entered their activity step counts as reported by their PA monitor for the prior 14 days (Phase 2: Days 15–28, occurred after enrollment).

At the end of the Phase 1 survey, participants were randomly assigned to receive one of two messages about Phase 2 (see Table 2). Those assigned to the high‐salience condition were informed that the study would include another brief survey two weeks from their initial survey completion date and that they would be required to input their daily step counts for the following 14 days. In contrast, participants assigned to the low‐salience condition were reminded to complete Phase 2 two weeks after being asked to report their daily steps for the past 14 days. Those in the low‐salience condition were further told that Phase 2 would be the same as Phase 1; thus, they could anticipate future observations of reporting their daily step counts, though this aspect of Phase 2 was not highlighted.

**TABLE 2:** Texts used for the saliency manipulation.

Condition	Text
Low saliency	You are almost done with this survey. On the next page, you will be asked to enter your name and banner ID number for course credit. Please remember that you will be asked to complete another survey in 2 weeks, which will ask the same questions as the current survey.
High saliency	You are almost done with this survey. On the next page, you will be asked to enter your name and banner ID number for course credit. Please remember that you will be asked to complete another survey in 2 weeks. This survey will ask you to enter your steps from your physical activity monitor for each day of that period.

### Measures

#### Demographics

Participants identified their age, gender, racial/ethnic background, most recent height and weight (for BMI calculation), marital status, and number of children.

#### PA monitor use and related characteristics

Participants were asked to identify the type of PA monitor they used (e.g., pedometer, wrist‐worn wearable, smartphone application) and the specific model (where applicable; e.g., Fitbit, Samsung, Apple). They were also asked to select the category that best described their PA monitor use with respect to length of time using it (1 = *1 month or less* to 6 = *more than 1 year*) and consistency of use (1 = *not at all* to 5 = *very*).

#### PA behavior

The behavioral outcome of interest was steps per day, as recorded by the participant's personal PA monitor. Steps per day is an indicator of overall movement and is more likely to reflect reactivity responses than PA at specific intensities; as noted, this metric is easier to modify (even without conscious intention) than activities at specific intensities (Cheval & Boisgontier, 2021). Further, as participants used a wide range of monitoring tools, it is the only metric that is available across all relevant tools (and for some tools, is the only metric collected). Participants were instructed to “Please look back through the last 14 days on your device/app and enter the total number of steps you took on each of the following days,” with numeric text boxes for “yesterday” (corresponding to Day 14), “2 days ago” (corresponding to Day 13), etc. in Phase 1. For Phase 2, “yesterday” corresponded to Day 28, “2 days ago” corresponded to Day 27, etc. We selected 14‐day periods for each to ensure adequate observation using commercially available wearables (Hilden et al., 2023). At each time point, participants were also asked to indicate whether they experienced illness, injury, stress, or other events that affected their PA behavior over the past two weeks (yes/no to each).

#### Social comparison orientation

To enable tests for individual differences in reactivity response based on SCO, participants were asked to complete the Iowa‐Netherlands Comparison Orientation Measure (Gibbons & Buunk, 1999) as part of the Phase 1 survey. This measure uses 11 items to assess respondents' agreement with statements such as “I always pay a lot of attention to how I do things compared with how others do things” on a scale of *strongly disagree* (1) to *strongly agree* (5). Numeric values for each response are summed to create a total score, with higher (vs. lower) total scores indicating stronger (vs. weaker) comparison orientation. This measure is frequently used to assess comparison orientation and consistently shows strong psychometric properties (Rose et al., 2024). In the present study, Cronbach's alpha was 0.75.

### Statistical approach

#### Exclusion of study participants and observations

Exclusion of survey responses was evaluated based on the following criteria: incompleteness, repetition of the same answers across multiple response sets, and unusual responses. Any responses that were deemed to be repeated, that did not offer a survey response in English, that included less than two valid days of data per survey, or where the condition was not recorded were removed from analyses. If a participant reported less than 100 steps on a given day or stated that they did not track steps on a given day, the day was set to missing (Bassett et al., 2017), as were vague responses (e.g., “5 k”). In terms of an upper limit of steps, as no guidelines exist, and adults in the U.S. who own PA monitors are known to have more positive attitudes toward PA and exercise and engage in more PA than non‐users (Dhingra et al., 2023), thus no outliers were excluded. In total, 69 observations were missing for Phase 1, and 44 observations were missing for Phase 2, out of a potential 3,528 observations per phase.

#### Data analysis

Data and code used in the present analysis are available from the Open Science Framework (https://osf.io/akvc9/). Multilevel regression analyses were conducted in R using lme4 (Bates et al., 2024) and lmertest (Kuznetsova et al., 2020); all models consisted of a within‐person level (level 1) and the between‐person level (level 2). For all models, the number of steps recorded per day was the outcome on level 1. As co‐registered (see https://osf.io/s2rby), we computed the following three models (including both fixed and random effects) to test whether study participation influenced the number of steps. Model assumptions were checked following the recommendations and scripts provided in Shaw and Flake (n.d.).

In the first model, we compared steps recorded before study participation (Days 1–14, as reported in Phase 1) to steps recorded while participating in the study (Days 15–28, as reported in Phase 2). In the second model, we compared steps recorded on the first day of participating in the study (Day 15, as reported in Phase 2) to all other days (Days 1–14 as reported in Phase 1, and Days 16–28 as reported in Phase 2). In the third model, we compared steps recorded on the first two days of participating in the study (Days 15–16, as reported in Phase 2) to the steps recorded on all other days (Days 1–14 as reported in Phase 1 and Days 17–28 as reported in Phase 2; see Maher et al., 2024). For each of the comparisons, dummy‐coded variables were used (1 = Day 15 or Days 15 and 16, depending on the model; 0 = all other days).

To test for the effects of the salience of researcher observation, we computed the same models including the condition (0 = low salience, 1 = high salience) as a predictor at level 2 and in cross‐level interactions with the previously entered predictor for the study phase. Sensitivity analyses for all models were computed to determine relevant covariates from BMI, length of time using the device (diverging from the co‐registration, this was dummy‐coded as one year or less vs more than one year due to the small number of responses for any category other than more than a year), age, and gender (all assessed on level 2). Because there were no meaningful changes in the results with the inclusion of covariates, we present the results from models with covariates in the Supplementary Material only. As a further sensitivity analysis, we computed the models only for participants who completed Phase 2 after exactly 14 days (*n* = 110). Again, as the results did not differ, these models are reported in the Supplementary Material. The level of statistical significance was set to 0.05 for all analyses. To facilitate interpretation, we computed semipartial correlations (*sr*) as a standardized measure of effect size (cf. Arigo et al., 2021; Baga et al., 2024). We also report differences in average steps per day as effect sizes.

Additionally, we conducted exploratory analyses that were not co‐registered: we examined the potential moderating effects of gender (dummy‐coded as 0 = woman, 1 = man), SCO (INCOM score, grand mean centered), and pre‐study PA level (mean steps for Days 1–14 as assessed in Phase 1, grand mean centered). These moderators were assessed on level 2 and were tested using cross‐level interactions. Simple slope analyses for pre‐study PA level were conducted with reghelper 1.1.2 (Hughes & Beiner, 2023).

## RESULTS

### Effects of study participation on steps

Intraclass correlations were comparable between phases (ICC_Phase 1_ = 0.51; ICC_Phase 2_ = 0.48), indicating that within‐person variability of steps did not meaningfully differ between phases.

#### Co‐registered analyses

Model results are summarized in Tables S2 (without covariates) and S3 (with covariates) in the supplementary material. The first model compared the steps per day reported for the 14 days prior to study participation (Phase 1, Days 1–14) to steps per day reported for the 14‐day study period (Phase 2, Days 15–28). Steps per day did not differ significantly between phases (*t*[250.6] = 0.86, *p* = 0.388, *sr* = 0.06). Participants reported an average of 8,087 steps (*SD* = 4,539) in Phase 1 and an average of 8,198 steps (*SD* = 4,518) in Phase 2, corresponding to a non‐significant difference of 111 steps per day.

Similarly, when comparing the steps recorded on the first day of study participation (Phase 2, Day 15) to all other days, there was no statistically significant difference (*t*[248.18] = 1.57, *p* = 0.117, *sr* = 0.08). There also was no statistically significant difference between steps per day recorded during the first two days of study participation (Phase 2, Days 15–16) to all other days (*t*[494.8] = −1.42, *p* = 0.157, *sr* = 0.07). However, when the latter predictor was included in the model, the effect for the first day of study participation reached statistical significance (*t*[2152.0] = 2.22, *p* = 0.027, *sr* = 0.09).

#### Exploratory analyses of moderators

Model results are summarized in Tables S4 (without covariates) and S5 (with covariates) in the supplementary material.

##### Gender

There was a significant cross‐level interaction for gender, indicating a moderating effect (*t*[242.6] = 3.76, *p* < 0.001, *srs* = 0.12). Compared to Phase 1 (Days 1–14, pre‐participation), women somewhat reduced their steps per day in Phase 2 (Days 15–28; from *M* = 7,937, *SD* = 4,385 to *M* = 7,756; *SD* = 4,125), while men increased their steps per day (from *M* = 8,623, *SD* = 4,812 to *M* = 9,548, *SD* = 5,228). Effects were comparable when specifically comparing steps per day recorded in Phase 1 to steps per day recorded on the first day of Phase 2 (Day 15; interaction effect: *t*[240.25] = 3.20, *p* = 0.001, *sr* = 0.15). However, in the model focusing on the first two days of study participation (Days 15–16), the interaction effect was no longer statistically significant (*t*[242.4] = 1.67, *p* = 0.096, *sr* = 0.08).

##### Social comparison orientation

There were no statistically significant interactions with SCO in any of the three models (model 1: *t*[248.31] = −0.12, *p* = .91, *sr* = 0.02; model 2: *t*[246.87] = −0.26, *p* = .798, *sr* = 0.03; model 3: *t*[251.04] = −0.36, *p* = .361, *sr* = 0.04).

##### PA during the pre‐study period

The interaction model controlled for a significant main effect of the average steps per day during the pre‐study period (*t*[6672.59] = 61.93, *p* < 0.001, *sr* = 0.50) showed a significant interaction of this predictor with the study phase for the first model (comparing steps per day reported in Phase 1 vs Phase 2; *t*[172.27] = −4.05, *p* < 0.001, *sr* = 0.13). A simple slope analysis (see also Table S6 in the supplementary material) indicated that at ‐1SD in steps in the pre‐study period, participants significantly increased their steps in Phase 2 (*b* = 623.61, *t*[172.93] = 3.43, *p* < .001), while participants with +1SD in steps in the pre‐study period significantly decreased their steps in Phase 2 (*b* = −419.05, *t*[171.28] = −2.29, *p* = .023). Similar interaction effects were observed for comparing steps recorded in Phase 1 to the steps per day recorded on the first day of study participation (Phase 2, Day 15; interaction: *t*[246.46] = −2.26, *p* = 0.024, *sr* = 0.12), although simple slopes were only statistically significant at ‐1SD (*b* = 855.83, *t*[246.76] = 2.73, *p* = .007), and the first two days of study participation (Phase 2, Days 15–16; interaction: *t*[249.10] = −2.47, *p* = 0.013, *sr* = 0.10), where simple slopes were only statistically significant at +1SD (*b* = −743.56, *t*[392.21] = −2.66, *p* = .008).

### Effects of the saliency of researcher observation

As noted, condition was introduced as a predictor on level 2, as was the interaction between condition and the main predictors of interest in the three models. Model results are summarized in Tables S7 (without covariates) and S8 (with covariates) in the supplementary material. Differences in steps per day between phases did not differ significantly depending on the salience condition (*t*[249.69] = 0.92, *p* = 0.360, *sr* = 0.06. In the low salience condition, participants reported *M* = 8,021 (*SD* = 4,621) steps per day in Phase 1 and *M* = 8,053 (*SD* = 4,666) steps per day in Phase 2, or an average non‐significant increase of 32 steps per day. In the high salience condition, participants reported *M* = 8,158 (*SD* = 4,450) steps per day in Phase 1 and *M* = 8,353 (*SD* = 4,350) steps per day in Phase 2, or an average non‐significant increase of 195 steps per day. Similar results were obtained when comparing the steps recorded in Phase 1 (Days 1–14, prior to participation) to steps per day recorded on the first day of study participation (Day 15; *t*[247.12] = −0.43, *p* = 0.665, *sr* = 0.04) and to steps per day recorded on the first two days of study participation (Days 15–16; *t*[251.36] = −0.06, *p* = 0.951, *sr* = 0.02).

## DISCUSSION

Findings from the present experimental two‐part study, which harnessed the unique benefits of existing data from experienced PA monitor users, indicate that the introduction of researcher observation induces only very small changes in PA behavior. These changes are unlikely to have practical significance for estimates of PA behavior in this population. With respect to PA behavior, measurement reactivity effects might thus be somewhat smaller than previously estimated (König et al., 2022), including in populations that have prior experience with PA self‐monitoring. As this population comprises approximately 35% of adults in the U.S. (Stewart, 2023) whose consumer behavior demonstrates an interest in PA self‐monitoring, a substantial proportion of participants in PA research studies likely have prior experience with PA monitoring devices and related behaviors.

Yet, empirical reports of research on reactivity to device‐based measurement of PA typically do not report on participants' prior experience with PA monitors nor statistically control for potential effects. This oversight makes it difficult to judge whether results in previous studies could have been influenced by a potential difference between experienced and novice PA monitor users. The present study can only speak to potential differences indirectly, based on smaller than expected effect sizes; (quasi‐)experimental work is needed to empirically confirm these hypotheses. Still, as the present findings show that experienced PA monitor users do not exhibit meaningful reactivity to the introduction of research participation (i.e., change relative to their own behavior pre‐participation), their inclusion in PA research studies alongside non‐users is unlikely to bias conclusions. An important next step to draw strong conclusions in this area is to enroll and directly compare experienced PA monitor users to participants who do not have prior experience with PA monitoring.

Results further indicate that intentionally emphasizing researcher observation has only a minimal impact on participants' steps per day. Potentially important, however, is that average changes from Phase 1 to Phase 2 were nearly four times higher with emphasis: participants in the low‐salience condition descriptively increased their average step count by less than 50 steps per day, while participants in the high‐salience condition descriptively increased their step count by almost 200 steps per day. It is possible that neither of these manipulations were particularly effective, as they appeared in text at the end of the Phase 1 survey and participants may not have engaged in meaningful processing of information about researcher observation. Due to a lacking manipulation check, we are unable to statistically control for the potential effect of awareness; future work should therefore assess the extent to which participants were aware of researcher observation during the study period. For designs similar to those of the present study, this would need to occur at the end of Phase 2 (retrospectively), as checking immediately after the manipulation is likely to add further potency to the manipulation. Participants also did not have any direct interaction with researchers in this study, which may blunt the potential salience of researcher observation. Yet, it is also possible that the power of our “high‐salience” manipulation was modest, relative to the low‐salience condition, and that protocols that call even greater attention to researcher observation (e.g., with direct researcher interaction) may see stronger reactivity patterns than those observed here (cf. Vaisman et al., 2020). Additional work is needed to identify the tipping point for reactivity to researcher observation and the role of direct interaction on patterns of PA behavior. Overall, however, it seems that PA studies without direct researcher interaction, as in the present study, show little risk of prompting reactivity.

These findings initially seem to contradict prior research on reactivity to the device‐based measurement of PA, which shows small‐to‐medium effects (König et al., 2022). However, it is important to note that findings are heterogeneous, and several studies report statistically nonsignificant findings (e.g., Davis et al., 2016; Hilgenkamp et al., 2012; Klenk et al., 2019). Further, the present study did show a subset of small but noteworthy effect sizes (e.g., *sr*s 0.10–0.13), and findings are consistent with prior work on health behaviors that show little effect of researcher observation on nutrient intake self‐reports (Steffen et al., 2021), minimal effects of direct researcher observation on physician visits (i.e., researchers present in the room during visits; Goodwin et al., 2017), and no effects of different study descriptions on self‐reported alcohol consumption (McCambridge et al., 2019).

Also of note were findings associated with three potential moderators, which we conducted in an exploratory manner. Pre‐study levels of PA moderated the effect of introducing researcher observation. Indeed, participants with lower pre‐study PA levels tended to increase their step count after enrollment. It is likely that the context of participating in a study of PA with behavioral reports had more influence on participants with lower PA engagement, as these individuals might be more aware of the distance between their current and healthy levels of PA (and thus, more room to move toward socially desirable PA engagement; Arigo & König, 2024). This finding is in line with Poulton et al. (2019), who showed that reactivity effects are more prominent in high‐risk drinkers, potentially because they are aware of the need for change. At the same time, participants with relatively high PA levels somewhat decreased their activity throughout the study. While we cannot rule out that this could be a regression to the mean, an alternative explanation seems more likely: Participants with high PA levels in Phase 1 exceeded commonly communicated norms for daily steps (i.e., 10,000). Participation in the study may have emphasized that they are already doing very well and thus may have curbed their motivation to further increase, or even reduced their motivation to be active, which resulted in slight decreases of step counts. Overall, these effects were small in the present study, but point toward the potential benefit of controlling for baseline levels of the behavior of interest (when possible).

Interestingly, results for gender as a moderator point in the opposite direction of expectations. Prior work shows little evidence of gender differences; one study demonstrated that girls are more prone to behavioral changes due to wearing a PA monitor than boys (Ho et al., 2013), though related evidence documented greater compliance with PA‐related treatment protocols among men than women (Krukowski et al., 2013). In line with considerable evidence of gender differences in PA among U.S. adults (Caspersen et al., 2000; Finkel et al., 2018; Troiano et al., 2008), women in this study reported lower PA engagement than men overall, though men showed a stronger average increase in PA engagement after enrollment. It is important to note that because the sample in the present study already wore a PA monitor, women might have already achieved the maximum benefit associated with self‐monitoring.

Indeed, women experience more competing demands in daily life than men due to balancing career and caregiving responsibilities, which are major barriers to physical activity engagement that are less likely to impede men's PA behavior (Lee & Tang, 2015; Rokicka & Zajkowska, 2020). Men also tend to be more interested in competition than women (Urbig et al., 2021) and may have seen participation in the present study as a challenge to increase their PA, either explicitly or implicitly. However, general SCO did not differentiate participants with respect to reactivity. As there was no indication that researchers would compare participants' PA behavior to that of others, this possibility may not have been salient. Given the strong influence of social comparison opportunities on PA and other health behaviors (Arigo et al., 2020; Arigo et al., 2023; Arigo et al., 2024; Olander et al., 2013), however, future work should explore the possibility that minimal contextual cues toward comparison could influence reactivity patterns.

### Strengths, limitations, and additional future directions

Using existing data that participants collected with their own PA monitors prior to study enrollment allowed us to overcome specific limitations of prior studies on reactivity to the device‐based measurement of PA. Specifically, these studies either compared wearing different monitors which were distributed by the research team (within or between participants; e.g., Clemes et al., 2008) or tested for changes in the collected data over time (e.g., Arigo & König, 2024; Baumann et al., 2018; Maher et al., 2024), assuming an initial change in behavior that returns to “typical” levels throughout the study period. In contrast, the design of the present study actually allowed for comparing PA behavior during a study to behavior before the study's onset. This design also allowed us to isolate the role of researcher observation on PA behavior, separating it from reactivity to a measurement device; to our knowledge, this is the first time such an approach has been used to test for reactivity patterns.

Yet, the limitations of this study design are worth noting. Allowing participants to use their own PA monitoring device resulted in the heterogeneity of devices used, which may differ in the accuracy of determining step counts (Fuller et al., 2020). The accuracy of reported data might have been further compromised by a lack of access to the raw data and reliance on participants' correct input into the survey. Because devices such as apps collect only steps per day (vs. minutes of light activity or moderate‐to‐vigorous‐intensity exercise, sedentary time), we were also limited to this PA outcome to avoid restriction of eligibility to users of a small number of devices. Although steps per day are more likely to show reactivity patterns than minutes of activity at different intensities (as the latter requires more intention and opportunity to modify; König et al., 2022), collecting other PA metrics in future studies would be informative. We were also unable to determine device wear time with precision, as we did not have direct access to device data. It is possible that differences between steps in Phase 1 versus Phase 2 in this study were due to changes in wear time, rather than in PA behavior. Yet, we observed *increases* in PA from Phase 1 to Phase 2, indicating increased wear time after the onset of research participation and researcher observation. We contend that increased wear time may itself be a manifestation of measurement reactivity and that this possibility warrants additional investigation. Further, even with less restrictive eligibility criteria in this respect and the use of multiple sources of recruitment, it took three years to achieve a sample size that is modest compared to prior research and still suffered from limited racial and ethnic diversity.

In a related vein, participants in the present sample were fairly active and predominantly identified as white, women, and of high socioeconomic status. These demographics mirrored known use patterns for PA monitoring devices; users are more likely to be women (vs. men), active (vs. not), affluent (vs. not), and white (vs. identifying with a minoritized group; Chandrasekaran et al., 2020). Although our sample accurately represented the population of interest, it is possible that other groups could respond differently to the introduction of researcher observation. For example, reactivity was stronger among those in this study who started at lower (vs. higher) levels of PA and might be particularly strong among those at even lower levels (i.e., those who were not represented in large numbers in this study). Work in this area would benefit from additional resources to advertise and encourage participation, particularly among groups that are minoritized.

Although a sample of 252 participants is relatively large compared to many other studies on reactivity to the device‐based measurement of physical activity, reported effects were smaller than anticipated based on the latest meta‐analysis (König et al., 2022). Since this study used a novel paradigm to study measurement reactivity, formal a‐priori power analysis could not be conducted due to a lack of relevant parameter estimates. It is also important to note that about half of the participants included in our analyses returned to the second part of the study later than planned. Sensitivity analyses indicated that results did not change if only participants were included that completed both surveys exactly 14 days apart. However, statistical power was reduced for these analyses, and in the larger sample, it is possible that the “effect” of the manipulation was missed (e.g., if Days 15 and 16 were not reported). Researchers who apply this approach in future studies should aim for maximizing completion on time, by only allowing participants to complete the survey on the respective day. Retention could be promoted by emphasizing the importance of completing the Phase 2 survey on time (in Phase 1), and sending a reminder a few days before the Phase 2 survey is due to be completed.

In sum, the present study demonstrates that introducing researcher observation without introducing an unfamiliar measurement device may result in little if any, detectable reactivity response with respect to PA behavior. However, the inclusion of participants who have prior experience with PA self‐monitoring devices with those who have no prior experience is likely to blunt sample‐level reactivity patterns, as introducing researcher observation and the overall context of participation in a research study does little to change PA behavior in the former group. Researchers should continue to use multiple days of PA monitoring (Hilden et al., 2023) and check for evidence of reactivity in studies that assess PA behavior across multiple days (Maher et al., 2024), with attention to prior experience with self‐monitoring devices (vs. not), gender, and starting levels of PA.

## CONFLICT OF INTEREST STATEMENT

The authors declare no conflicts of interest.

## ETHICS APPROVAL STATEMENT

The study was approved by Rowan University's Institutional Review Board (approval number PRO‐2021‐379).

## PERMISSION TO REPRODUCE MATERIAL FROM OTHER SOURCES

Not applicable.

## CLINICAL TRIAL REGISTRATION

The analysis was coregistered on the Open Science Framework (https://osf.io/s2rby/).

## Supporting information


**Table S1.** Sociodemographic characteristics by recruitment modality (introductory psychology classes vs. online advertisements).
**Table S2.** Pre‐registered fixed effects models for steps (*N* = 252).
**Table S3.** Pre‐registered fixed effects models for steps, controlled for gender, age, BMI, and tracker use duration (*N* = 235).
**Table S4.** Fixed effects models for exploratory analyses of moderators (*N* = 252).
**Table S5.** Fixed effects models for exploratory analyses of moderators, controlled for gender, age, BMI, and tracker use duration.
**Table S6.** Results of simple slopes analysis for testing physical activity during Phase 1 as a moderator.
**Table S7.** Fixed effects models testing the effects of the saliency of researcher observation (*N* = 252).
**Table S8.** Fixed effects models testing the effects of the saliency of researcher observation, controlled for gender, age, BMI, and tracker use duration (*N* = 235).
**Table S9.** Fixed effects models for steps; only for participants who completed the questionnaires exactly 14 days apart (*N* = 110).
Table S10. Fixed effects models for exploratory analyses of moderators; only for participants who completed the questionnaires exactly 14 days apart.
**Table S11.** Fixed effects models testing the effects of saliency of researcher observation; only for participants who completed the questionnaires exactly 14 days apart (*N* = 110).
**Table S12.** Fixed effects models for steps with covariates; only for participants who completed the questionnaires exactly 14 days apart, controlled for gender, age, BMI, and tracker use duration (*N* = 105).
**Table S13.** Fixed effects models for exploratory analyses of moderators with covariates; only for participants who completed the questionnaires exactly 14 days apart, controlled for gender, age, BMI, and tracker use duration.
**Table S14.** Fixed effects models testing the effects of saliency of researcher observation with covariates; only for participants who completed the questionnaires exactly 14 days apart, controlled for gender, age, BMI, and tracker use duration (*N* = 105).

## Data Availability

Data and code are available from the project's Open Science Framework page (https://osf.io/akvc9/).

## ENDNOTE

1 See Table S1 in the online supplement for a comparison of demographic characteristics between the two subsamples.
