# Supplementary material for: Isolating the role of researcher observation on reactivity to the measurement of physical activity
Source: Appl Psychol Health Well Being. 2024 Dec 19;17(1):e12630. doi: 10.1111/aphw.12630 (PMC11659718; doi:10.1111/aphw.12630)
Supplement: Supplementary file 1 — Table S1. Sociodemographic characteristics by recruitment modality (introductory psychology classes vs. online advertisements). Table S2. Pre‐registered fixed effects models for steps (N = 252). Table S3. Pre‐registered fixed effects models for steps, controlled for gender, age, BMI, and tracker use duration (N = 235). Table S4. Fixed effects models for exploratory analyses of moderators (N = 252). Table S5. Fixed effects models for exploratory analyses of moderators, controlled for gender, age, BMI, and tracker use duration. Table S6. Results of simple slopes analysis for testing physical activity during Phase 1 as a moderator. Table S7. Fixed effects models testing the effects of the saliency of researcher observation (N = 252). Table S8. Fixed effects models testing the effects of the saliency of researcher observation, controlled for gender, age, BMI, and tracker use duration (N = 235). Table S9. Fixed effects models for steps; only for participants who completed the questionnaires exactly 14 days apart (N = 110). Table S10. Fixed effects models for exploratory analyses of moderators; only for participants who completed the questionnaires exactly 14 days apart. Table S11. Fixed effects models testing the effects of saliency of researcher observation; only for participants who completed the questionnaires exactly 14 days apart (N = 110). Table S12. Fixed effects models for steps with covariates; only for participants who completed the questionnaires exactly 14 days apart, controlled for gender, age, BMI, and tracker use duration (N = 105). Table S13. Fixed effects models for exploratory analyses of moderators with covariates; only for participants who completed the questionnaires exactly 14 days apart, controlled for gender, age, BMI, and tracker use duration. Table S14. Fixed effects models testing the effects of saliency of researcher observation with covariates; only for participants who completed the questionnaires exactly 14 days apart, controlled for gender [file APHW-17-0-s001.docx]

SUPPLEMENT

Table S1. Sociodemographic characteristics by recruitment modality (introductory psychology classes vs. online advertisements).

|  | Response options | Recruited via introductory psychology classes (*n* = 81) | Recruited online (n = 172) | Comparison of recruitment modalities |
| --- | --- | --- | --- | --- |
| Age (M, SD) |  | 19.51, 2.96 | 35.34, 11.92 | *t*(208.81) = -16.18, *p* < .001 |
| Gender (%) | Women | 70.4% | 71.3% | Χ²(*df* = 4) = 6.64, *p* = .156 |
|  | Men | 27.2% | 25.7% |  |
|  | Gender-neutral | 0.0% | 0.6% |  |
|  | Non-binary | 2.5% | 2.3% |  |
|  | Would rather not say |  | 0.0% |  |
| Ethnic or racial heritage (%) | American Indian or Alaska Native | 1.2% | 0.0% | Χ²(*df* = 5) = 6.75, *p* = .240 |
|  | East Asian | 2.5% | 2.4% |  |
|  | Black or African American | 13.6% | 5.9% |  |
|  | White | 71.6% | 80.6% |  |
|  | Multiracial | 3.7% | 4.7% |  |
|  | Other | 7.4% | 6.5% |  |
| Ethnicity (%) | Not Hispanic or Latinx | 84.0% | 91.2% | Χ²(*df* = 1) = 2.89, *p* = .089 |
|  | Hispanic or Latinx | 16.0% | 8.8% |  |
| BMI (M, SD) |  | 24.46, 5.49 | 26.49, 6.31 | *t*(242) = -2.47, *p* = .014 |
| Physical activity tracking device (%) | Watch/Wrist band | 49.4% | 82.5% | Χ²(*df* = 5) = 38.71, *p* < .001 |
|  | Ring/ Smart jewlry | 0.0% | 1.2% |  |
|  | Smart clothing | 0.0% | 0.6% |  |
|  | Pedometer | 1.2% | 0.0% |  |
|  | Phone application | 46.9% | 12.9% |  |
|  | Other | 2.5% | 2.9% |  |
| Tracker use duration (%) | 1 month or less | 12.5% | 4.7% | Χ²(*df* = 5) = 8.39, *p* = .136 |
|  | 2-3 months | 6.3% | 5.3% |  |
|  | 4-5 months | 7.5% | 3.5% |  |
|  | 6 months | 5.0% | 3.5% |  |
|  | 6 months – 1 year | 7.5% | 10.5% |  |
|  | More than 1 year | 61.3% | 72.5% |  |
| Use consistency (M, SD) |  | 4.89, 1.25 | 5.63, 0.69 | *t*(103.67) = -4.98, *p* < .001 |
| Average steps for pre-study period, reported in Phase 1 |  | 7514.05, 3117.03 | 8321.33, 3402.04 | *t*(249) = -1.81, *p* = .072 |
| Median of steps for pre-study period, reported in Phase 1 |  | 7266.14 | 7654.65 | *-* |
| Physical activity levels typical in past 14 days, reported in Phase 1 | No  Yes | 11.1%  88.9% | 20.5%  79.5% | Χ²(*df* = 1) = 3.34, *p* = .068 |
| Physical activity levels typical in past 14 days, reported in Phase 2 | No  Yes | 17.3%  82.7% | 25.1%  74.9% | Χ²(*df* = 1) = 1.94, *p* = .164 |
| Covid-19 reported in Phase 1 | No  Yes | 98.8%  1.2% | 99.4%  0.6% | Χ²(*df* = 1) = 0.30, *p* = .587 |
| Covid-19, reported in Phase 2^1^ | No  Yes | 100.0%  0.0% | 100.0%  0.0% | - |
| Other respiratory illness, reported in Phase 1 | No  Yes | 91.4%  8.6% | 94.2%  5.8% | Χ²(*df* = 1) = 0.68, *p* = .409 |
| Other respiratory illness, reported in Phase 2 | No  Yes | 87.7%  12.3% | 92.4%  7.6% | Χ²(*df* = 1) = 1.46, *p* = .228 |

Table S2. Pre-registered fixed effects models for steps (*N* = 252).

|  | Model 1 | | | | | | Model 2 | | | | | | Model 3 | | | | | |
| --- | --- | --- | --- | --- | --- | --- | --- | --- | --- | --- | --- | --- | --- | --- | --- | --- | --- | --- |
|  | b | SE | t | df | p | srs | b | SE | t | df | p | srs | b | SE | t | df | p | srs |
| Intercept | 8076.88 | 209.45 | 38.56 | 249.6 | <0.001 |  | 8077.82 | 209.47 | 38.56 | 249.57 | <0.001 |  | 8077.69 | 209.46 | 38.56 | 249.6 | <0.001 |  |
| Days 1-14 vs Days 15-28 | 105.53 | 122.26 | 0.86 | 250.6 | 0.388 | 0.06 | 79.53 | 120.38 | 0.66 | 250.70 | 0.509 | 0.05 | 104.21 | 121.36 | 0.86 | 250.7 | 0.391 | 0.06 |
| Day 15 vs all other days |  |  |  |  |  |  | 349.80 | 223.32 | 1.57 | 248.18 | 0.117 | 0.08 | 637.66 | 287.44 | 2.22 | 2152.2 | 0.027 | 0.09 |
| Days 15-16 vs all other days |  |  |  |  |  |  |  |  |  |  |  |  | -311.78 | 220.26 | -1.42 | 494.8 | 0.157 | 0.07 |

Table S3. Pre-registered fixed effects models for steps, controlled for gender, age, BMI, and tracker use duration (*N* = 235).

|  | Model 1 | | | | | | Model 2 | | | | | | Model 3 | | | | | |
| --- | --- | --- | --- | --- | --- | --- | --- | --- | --- | --- | --- | --- | --- | --- | --- | --- | --- | --- |
|  | b | SE | t | df | p | srs | b | SE | t | df | p | srs | b | SE | t | df | p | srs |
| Intercept | 8290.36 | 986.08 | 8.41 | 228.83 | <0.001 |  | 8220.19 | 977.87 | 8.41 | 229.05 | <0.001 |  | 8216.37 | 977.93 | 8.40 | 229.03 | <0.001 |  |
| Gender: women vs men | 1659.48 | 471.66 | 3.52 | 227.11 | <0.001 | 0.12 | 1780.50 | 467.52 | 3.81 | 226.91 | <0.001 | 0.13 | 1764.58 | 467.57 | 3.77 | 226.94 | <0.001 | 0.13 |
| Gender: women vs gender neutral | -3884.36 | 3093.02 | -1.26 | 226.98 | 0.209 | 0.07 | -3266.26 | 3064.47 | -1.07 | 226.36 | 0.287 | 0.07 | -3391.02 | 3064.60 | -1.11 | 226.33 | 0.269 | 0.07 |
| Gender: women vs non-binary | 340.26 | 1565.76 | 0.22 | 226.25 | 0.828 | 0.03 | 468.01 | 1551.96 | 0.30 | 226.01 | 0.763 | 0.04 | 471.98 | 1552.10 | 0.30 | 226.02 | 0.761 | 0.04 |
| Gender: women vs would rather not say | -1184.78 | 2202.69 | -0.54 | 226.23 | 0.591 | 0.05 | -1008.90 | 2183.27 | -0.46 | 225.99 | 0.644 | 0.04 | -1029.29 | 2183.46 | -0.47 | 226.00 | 0.637 | 0.04 |
| Age | 66.17 | 17.11 | 3.87 | 226.71 | <0.001 | 0.13 | 63.98 | 16.97 | 3.77 | 226.61 | <0.001 | 0.13 | 64.33 | 16.97 | 3.79 | 226.61 | <0.001 | 0.13 |
| BMI | -102.27 | 34.77 | -2.94 | 226.77 | 0.003 | 0.11 | -101.52 | 34.47 | -2.95 | 226.63 | 0.003 | 0.11 | -101.14 | 34.47 | -2.93 | 226.64 | 0.003 | 0.11 |
| Tracker use duration^1^ | 11.19 | 449.95 | 0.02 | 227.10 | 0.980 | 0.01 | 128.88 | 446.04 | 0.29 | 226.97 | 0.773 | 0.04 | 111.64 | 446.08 | 0.25 | 226.98 | 0.802 | 0.03 |
| Days 1-14 vs Days 15-28 | 107.91 | 126.49 | 0.85 | 233.89 | 0.394 | 0.06 | 82.51 | 125.05 | 0.66 | 234.02 | 0.509 | 0.05 | 108.12 | 125.99 | 0.86 | 234.07 | 0.391 | 0.06 |
| Day 15 vs all other days |  |  |  |  |  |  | 339.73 | 231.73 | 1.47 | 231.07 | 0.143 | 0.08 | 636.89 | 298.57 | 2.13 | 2599.02 | 0.033 | 0.10 |
| Days 15-16 vs all other days |  |  |  |  |  |  |  |  |  |  |  |  | -321.86 | 232.62 | -1.38 | 419.78 | .167 | 0.08 |

Note. ^1^ Tracker use duration was dummy-coded for this analysis (0 = one year or less, 1 = more than a year).

Table S4. Fixed effects models for exploratory analyses of moderators (*N* = 252).

|  | Model 1 | | | | | | Model 2 | | | | | | Model 3 | | | | | |
| --- | --- | --- | --- | --- | --- | --- | --- | --- | --- | --- | --- | --- | --- | --- | --- | --- | --- | --- |
|  | b | SE | t | df | p | srs | b | SE | t | df | p | srs | b | SE | t | df | p | srs |
| ***Gender*** | | | | | | | | | | | | | | | | | | |
| Intercept | 7896.75 | 246.25 | 32.07 | 241.8 | <0.001 |  | 7773.63 | 244.35 | 31.81 | 248.39 | <0.001 |  | 7769.03 | 244.52 | 31.77 | 247.9 | <0.001 |  |
| Days 1-14 vs Days 15-28 | -158.88 | 139.74 | -1.14 | 242.4 | 0.256 | 0.07 | 89.65 | 121.03 | 0.74 | 243.75 | 0.460 | 0.05 | 114.89 | 121.84 | 0.94 | 243.8 | 0.346 | 0.06 |
| Gender^1^ | 780.80 | 475.09 | 1.64 | 242.7 | 0.100 | 0.08 | 1248.60 | 456.42 | 2.74 | 242.79 | 0.007 | 0.11 | 1265.18 | 457.58 | 2.76 | 242.8 | 0.006 | 0.11 |
| Days 1-14 vs Days 15-28 * Gender | 1017.75 | 270.45 | 3.76 | 242.6 | <0.001 | 0.12 | -12.99 | 264.17 | -0.05 | 240.13 | 0.958 |  |  |  |  |  |  |  |
| Day 15 vs all other days |  |  |  |  |  |  | -12.99 | 264.17 | -0.05 | 240.13 | 0.958 | 0.01 | 601.93 | 289.56 | 2.08 | 2412.9 | 0.038 | 0.09 |
| Day 15 vs all other days * Gender |  |  |  |  |  |  | 1194.55 | 506.03 | 2.36 | 240.22 | 0.019 | 0.10 |  |  |  |  |  |  |
| Days 15-16 vs all other days |  |  |  |  |  |  |  |  |  |  |  |  | -497.02 | 248.95 | -2.00 | 417.2 | 0.046 | 0.09 |
| Days 15-16 vs all other days * Gender |  |  |  |  |  |  |  |  |  |  |  |  | 669.29 | 401.44 | 1.67 | 242.4 | 0.096 | 0.08 |
| ***SCO*** | | | | | | | | | | | | | | | | | | |
| Intercept | 8076.89 | 208.82 | 38.68 | 248.68 | <0.001 |  | 8077.89 | 208.82 | 38.68 | 248.78 | <0.001 |  | 8077.75 | 208.82 | 38.68 | 248.80 | <0.001 |  |
| Days 1-14 vs Days 15-28 | 105.52 | 122.49 | 0.86 | 249.62 | 0.389 | 0.06 | 79.47 | 120.37 | 0.66 | 250.75 | 0.509 | 0.05 | 104.15 | 121.35 | 0.86 | 250.76 | 0.391 | 0.06 |
| SCO | -47.70 | 30.01 | -1.59 | 247.99 | 0.112 | 0.08 | -48.36 | 29.00 | -1.67 | 249.06 | 0.095 | 0.08 | -48.03 | 29.06 | -1.65 | 249.00 | 0.098 | 0.08 |
| Days 1-14 vs Days 15-28 * SCO | -2.06 | 17.56 | -0.12 | 248.31 | 0.907 | 0.02 |  |  |  |  |  |  |  |  |  |  |  |  |
| Day 15 vs all other days |  |  |  |  |  |  | 350.47 | 223.74 | 1.57 | 247.13 | 0.117 | 0.8 | 6927.00 | 638.26 | 287.43 | 2158.40 | 0.026 | 0.09 |
| Day 15 vs all other days * SCO |  |  |  |  |  |  | -8.22 | 32.03 | -0.26 | 246.87 | 0.797 | 0.03 |  |  |  |  |  |  |
| Days 15-16 vs all other days |  |  |  |  |  |  |  |  |  |  |  |  | -311.75 | 220.46 | -1.41 | 489.92 | 0.157 | 0.07 |
| Days 15-16 vs all other days * SCO |  |  |  |  |  |  |  |  |  |  |  |  | -9.08 | 25.13 | -0.36 | 251.04 | 0.718 | 0.04 |
| ***PA during the pre-study period*** | | | | | | | | | | | | | | | | | | |
| Intercept | 8052.12 | 53.23 | 151.26 | 6672.59 | <0.001 |  | 8052.96 | 53.06 | 151.78 | 6348.56 | <0.001 |  | 8052.89 | 52.84 | 152.39 | 6341.25 | <0.001 |  |
| Days 1-14 vs Days 15-28 | 106.31 | 128.94 | 0.82 | 172.69 | 0.410 | 0.13 | 78.93 | 130.32 | 0.61 | 272.39 | 0.545 | 0.12 | 103.53 | 131.26 | 0.79 | 276.31 | 0.430 | 0.06 |
| PA pre-study period | 0.99 | 0.02 | 61.93 | 6672.59 | <0.001 | 0.50 | 0.97 | 0.01 | 66.68 | 903.40 | <0.001 | 0.50 | 0.97 | 0.01 | 66.98 | 988.52 | <0.001 | 0.52 |
| Days 1-14 vs Days 15-28 * PA pre-study period | -0.16 | 0.04 | -4.05 | 172.27 | <0.001 | 0.13 |  |  |  |  |  |  |  |  |  |  |  |  |
| Day 15 vs all other days |  |  |  |  |  |  | 359.12 | 222.15 | 1.62 | 246.29 | 0.106 | 0.08 | 644.47 | 282.72 | 2.28 | 2206.89 | 0.023 | 0.10 |
| Day 15 vs all other days * PA pre-study period |  |  |  |  |  |  | -0.15 | 0.07 | -2.26 | 246.44 | 0.024 | 0.12 |  |  |  |  |  |  |
| Days 15-16 vs all other days |  |  |  |  |  |  |  |  |  |  |  |  | -309.63 | 218.73 | -1.42 | 438.65 | 0.157 | 0.08 |
| Days 15-16 vs all other days * PA pre-study period |  |  |  |  |  |  |  |  |  |  |  |  | -0.13 | 0.05 | -2.47 | 249.10 | 0.013 | 0.10 |

Note. ^1^ Gender was dummy-coded for this analysis (0 = women, 1 = men).

Table S5. Fixed effects models for exploratory analyses of moderators, controlled for gender, age, BMI, and tracker use duration.

|  | Model 1 | | | | | | Model 2 | | | | | | Model 3 | | | | | |
| --- | --- | --- | --- | --- | --- | --- | --- | --- | --- | --- | --- | --- | --- | --- | --- | --- | --- | --- |
|  | b | SE | t | df | p | srs | b | SE | t | df | p | srs | b | SE | t | df | p | srs |
| ***Gender^1^ (N = 228)*** | | | | | | | | | | | | | | | | | | |
| Intercept | 8213.50 | 993.42 | 8.27 | 225.19 | <0.001 |  | 8068.87 | 984.84 | 8.19 | 225.04 | <0.001 |  | 8047.37 | 984.56 | 8.17 | 225.09 | <0.001 |  |
| Age | 67.79 | 17.16 | 3.95 | 222.74 | <0.001 | 0.13 | 65.38 | 17.01 | 3.84 | 222.61 | <0.001 | 0.13 | 65.69 | 17.00 | 3.86 | 222.62 | <0.001 | 0.13 |
| BMI | -95.96 | 34.94 | -2.75 | 222.82 | 0.006 | 0.11 | -95.63 | 34.64 | -2.76 | 222.64 | 0.006 | 0.11 | -94.97 | 34.63 | -2.74 | 222.66 | 0.006 | 0.11 |
| Tracker use duration^2^ | 18.85 | 455.07 | 0.04 | 223.16 | 0.967 | 0.01 | 140.55 | 451.15 | 0.31 | 222.99 | 0.755 | 0.04 | 124.26 | 451.01 | 0.28 | 223.02 | 0.783 | 0.04 |
| Days 1-14 vs Days 15-28 | -154.10 | 143.35 | -1.08 | 225.43 | 0.282 | 0.07 | 94.25 | 125.80 | 0.75 | 226.93 | 0.454 | 0.06 | 120.46 | 126.52 | 0.95 | 226.81 | 0.341 | 0.06 |
| Gender^3^ | 1089.80 | 494.34 | 2.20 | 224.75 | 0.028 | 0.10 | 1583.75 | 473.97 | 3.34 | 223.37 | 0.001 | 0.12 | 1608.86 | 474.80 | 3.39 | 223.64 | 0.001 | 0.12 |
| Days 1-14 vs Days 15-28 * Gender | 1056.90 | 283.33 | 3.73 | 225.87 | <0.001 | 0.13 |  |  |  |  |  |  |  |  |  |  |  |  |
| Day 15 vs all other days |  |  |  |  |  |  | -32.62 | 272.60 | -0.12 | 223.10 | 0.905 | 0.02 | 598.22 | 300.68 | 1.99 | 3567.13 | 0.047 | 0.09 |
| Day 15 vs all other days * Gender |  |  |  |  |  |  | 1265.32 | 532.48 | 2.38 | 223.13 | 0.018 | 0.10 |  |  |  |  |  |  |
| Days 15-16 vs all other days |  |  |  |  |  |  |  |  |  |  |  |  | -527.44 | 265.35 | -1.99 | 432.02 | 0.047 | 0.09 |
| Days 15-16 vs all other days * Gender |  |  |  |  |  |  |  |  |  |  |  |  | 774.79 | 431.06 | 1.80 | 219.88 | 0.072 | 0.09 |
| ***SCO (N = 235)*** | | | | | | | | | | | | | | | | | | |
| Intercept | 8345.60 | 994.93 | 8.39 | 227.84 | <0.001 |  | 8277.88 | 986.62 | 8.39 | 228.05 | <0.001 |  | 8275.46 | 986.61 | 8.39 | 228.07 | <0.001 |  |
| Gender: women vs men | 1633.97 | 475.78 | 3.43 | 226.14 | 0.001 | 0.12 | 1754.18 | 471.62 | 3.72 | 225.96 | <0.001 | 0.13 | 1737.17 | 471.63 | 3.68 | 226.01 | <0.001 | 0.13 |
| Gender: women vs gender neutral | -3882.70 | 3098.37 | -1.25 | 226.00 | 0.210 | 0.07 | -3263.14 | 3069.71 | -1.06 | 225.38 | 0.288 | 0.07 | -3390.03 | 3069.61 | -1.10 | 225.38 | 0.269 | 0.07 |
| Gender: women vs non-binary | 346.15 | 1568.52 | 0.22 | 225.27 | 0.825 | 0.03 | 474.46 | 1554.66 | 0.31 | 225.03 | 0.760 | 0.04 | 478.81 | 1554.68 | 0.31 | 225.07 | 0.758 | 0.04 |
| Gender: women vs would rather not say | -1185.30 | 2206.51 | -0.54 | 225.25 | 0.591 | 0.05 | -1009.20 | 2187.00 | -0.46 | 225.01 | 0.644 | 0.04 | -1029.89 | 2187.04 | -0.47 | 225.05 | 0.638 | 0.04 |
| Age | 63.46 | 18.11 | 3.50 | 225.81 | <0.001 | 0.12 | 61.13 | 17.95 | 3.41 | 225.88 | 0.001 | 0.12 | 61.41 | 17.95 | 3.42 | 225.83 | 0.001 | 0.12 |
| BMI | -101.12 | 34.92 | -2.90 | 225.79 | 0.004 | 0.11 | -100.31 | 34.61 | -2.90 | 225.66 | 0.004 | 0.11 | -99.88 | 34.61 | -2.89 | 225.70 | 0.004 | 0.11 |
| Tracker use duration^2^ | 17.06 | 450.90 | 0.04 | 226.11 | 0.970 | 0.01 | 135.31 | 446.97 | 0.30 | 225.98 | 0.762 | 0.04 | 117.95 | 446.98 | 0.26 | 226.02 | 0.792 | 0.03 |
| Days 1-14 vs Days 15-28 | 107.93 | 126.76 | 0.85 | 232.90 | 0.395 | 0.06 | 82.51 | 125.04 | 0.66 | 234.03 | 0.509 | 0.05 | 108.13 | 125.99 | 0.86 | 234.07 | 0.391 | 0.06 |
| SCO | -13.69 | 32.38 | -0.42 | 228.15 | 0.672 | 0.04 | -14.97 | 30.97 | -0.48 | 226.60 | 0.629 | 0.05 | -13.74 | 31.00 | -0.44 | 227.11 | 0.658 | 0.04 |
| Days 1-14 vs Days 15-28 * SCO | -1.16 | 18.26 | -0.06 | 231.66 | 0.949 | 0.02 |  |  |  |  |  |  |  |  |  |  |  |  |
| Day 15 vs all other days |  |  |  |  |  |  | 339.94 | 232.24 | 1.46 | 230.16 | 0.143 | 0.08 | 637.30 | 298.57 | 2.13 | 2601.29 | 0.033 | 0.10 |
| Day 15 vs all other days * SCO |  |  |  |  |  |  | -0.01 | 33.44 | -0.00 | 229.92 | 1.000 | 0.00 |  |  |  |  |  |  |
| Days 15-16 vs all other days |  |  |  |  |  |  |  |  |  |  |  |  | -322.02 | 232.86 | -1.38 | 416.20 | 0.167 | 0.08 |
| Days 15-16 vs all other days * SCO |  |  |  |  |  |  |  |  |  |  |  |  | -8.03 | 26.73 | -0.30 | 230.44 | 0.764 | 0.04 |
| ***PA during the pre-study period (N = 234)*** | | | | | | | | | | | | | | | | | | |
| Intercept | 8052.04 | 248.65 | 32.38 | 49.77 | <0.001 |  | 8057.04 | 249.22 | 32.33 | 3200.77 | <0.001 |  | 8056.86 | 248.11 | 32.47 | 1209.06 | <0.001 |  |
| Gender: women vs men | 241.82 | 119.86 | 2.02 | 44.89 | 0.044 | 0.09 | 216.08 | 120.16 | 1.80 | 3062.65 | 0.072 | 0.09 | 215.94 | 119.63 | 1.80 | 1116.69 | 0.071 | 0.09 |
| Gender: women vs gender neutral | -60.44 | 771.50 | -0.08 | 45.63 | 0.938 | 0.02 | -111.70 | 773.44 | -0.14 | 3151.61 | 0.885 | 0.02 | -111.85 | 770.02 | -0.15 | 1139.36 | 0.885 | 0.03 |
| Gender: women vs non-binary | 55.11 | 389.22 | 0.14 | 44.43 | 0.887 | 0.02 | 38.43 | 390.18 | 0.10 | 3063.60 | 0.922 | 0.02 | 38.77 | 388.46 | 0.10 | 1109.67 | 0.921 | 0.02 |
| Gender: women vs would rather not say | -158.16 | 547.43 | -0.29 | 44.41 | 0.773 | 0.04 | -164.83 | 548.78 | -0.30 | 3064.91 | 0.764 | 0.04 | -164.01 | 546.36 | -0.30 | 1109.54 | 0.764 | 0.04 |
| Age | 0.70 | 4.44 | 0.16 | 44.90 | 0.874 | 0.03 | 0.82 | 4.45 | 0.18 | 3045.88 | 0.854 | 0.03 | 0.81 | 4.43 | 0.18 | 1114.48 | 0.855 | 0.03 |
| BMI | -2.97 | 8.89 | -0.33 | 45.00 | 0.738 | 0.04 | -2.59 | 8.92 | -0.29 | 3040.42 | 0.771 | 0.04 | -2.59 | 8.88 | -0.29 | 1115.34 | 0.770 | 0.04 |
| Tracker use duration^2^ | -7.11 | 113.58 | -0.06 | 45.22 | 0.950 | 0.02 | -20.99 | 113.87 | -0.18 | 3038.93 | 0.854 | 0.03 | -20.56 | 113.37 | -0.18 | 1118.60 | 0.856 | 0.04 |
| Days 1-14 vs Days 15-28 | 112.39 | 132.07 | 0.85 | 44.23 | 0.395 | 0.06 | 82.83 | 133.73 | 0.62 | 321.31 | 0.536 | 0.05 | 108.36 | 134.65 | 0.80 | 282.02 | 0.421 | 0.06 |
| PA pre-study period | 0.99 | 0.02 | 57.50 | 3999.01 | <0.001 | 0.50 | 0.96 | 0.02 | 61.21 | 3059.84 | <0.001 | 0.51 | 0.97 | 0.02 | 61.52 | 1165.10 | <0.001 | 0.51 |
| Days 1-14 vs Days 15-28 * PA pre-study period | -0.16 | 0.04 | -4.14 | 44.24 | <0.001 | 0.13 |  |  |  |  |  |  |  |  |  |  |  |  |
| Day 15 vs all other days |  |  |  |  |  |  | 354.20 | 229.98 | 1.54 | 228.96 | 0.124 | 0.08 | 645.54 | 293.39 | 2.20 | 2891.90 | 0.028 | 0.10 |
| Day 15 vs all other days * PA pre-study period |  |  |  |  |  |  | -0.17 | 0.07 | -2.55 | 229.25 | 0.011 | 0.10 |  |  |  |  |  |  |
| Days 15-16 vs all other days |  |  |  |  |  |  |  |  |  |  |  |  | -317.04 | 231.42 | -1.37 | 378.10 | 0.171 | 0.08 |
| Days 15-16 vs all other days * PA pre-study period |  |  |  |  |  |  |  |  |  |  |  |  | -0.14 | 0.05 | -2.59 | 229.10 | 0.010 | 0.11 |

Note. ^1^ Since gender was introduced as a moderator, it was not introduced as a covariate in this model. ^2^ Tracker use duration was dummy-coded for this analysis (0 = one year or less, 1 = more than a year). ^3^ Gender was dummy-coded for this analysis (0 = women, 1 = men).

Table S6. Results of simple slopes analysis for testing physical activity during Phase 1 as a moderator.

| Physical activity (moderator) | Model 1 | | | | | Model 2 | | | | | Model 3 | | | | |
| --- | --- | --- | --- | --- | --- | --- | --- | --- | --- | --- | --- | --- | --- | --- | --- |
|  | b | SE | t | df | p | b | SE | t | df | p | b | SE | t | df | p |
| ***Without covariates*** | | | | | | | | | | | | | | | |
| -1 SD | 623.61 | 181.87 | 3.43 | 172.88 | < .001 | 855.83 | 313.47 | 2.73 | 246.73 | 0.007 | 117.76 | 279.64 | 0.42 | 394.18 | 0.674 |
| M | 128.94 | 171.97 | 0.79 | 171.97 | 0.429 | 355.25 | 222.14 | 1.60 | 246.24 | 0.111 | -312.95 | 218.72 | -1.43 | 438.66 | 0.153 |
| +1 SD | -419.05 | 182.69 | -2.29 | 171.29 | 0.023 | -145.27 | 314.49 | -0.46 | 246.13 | 0.645 | -743.38 | 279.40 | -2.66 | 392.12 | 0.008 |
| ***With covariates*** |  |  |  |  |  |  |  |  |  |  |  |  |  |  |  |
| -1 SD | 610.02 | 185.39 | 3.29 | 325.47 | 0.001 | 933.48 | 324.47 | 2.88 | 229.45 | 0.004 | 154.32 | 295.76 | 0.52 | 350.03 | 0.602 |
| M | 78.73 | 121.46 | 0.60 | 322.81 | 0.550 | 346.85 | 229.98 | 1.51 | 228.97 | 0.132 | -323.02 | 231.41 | -1.40 | 378.07 | 0.164 |
| +1 SD | -452.55 | 186.29 | -2.43 | 321.27 | 0.016 | -239.78 | 325.68 | -0.74 | 228.85 | 0.462 | -799.37 | 295.55 | -2.71 | 348.26 | 0.007 |

Table S7. Fixed effects models testing the effects of saliency of researcher observation (*N* = 252).

|  | Model 1 | | | | | | Model 2 | | | | | | Model 3 | | | | | |
| --- | --- | --- | --- | --- | --- | --- | --- | --- | --- | --- | --- | --- | --- | --- | --- | --- | --- | --- |
|  | b | SE | t | df | p | srs | b | SE | t | df | p | srs | b | SE | t | df | p | srs |
| Intercept | 8052.85 | 292.25 | 27.55 | 248.38 | <0.001 |  | 8002.56 | 287.43 | 27.84 | 257.39 | <0.001 |  | 8009.81 | 287.72 | 27.84 | 256.83 | <0.001 |  |
| Condition^1^ | 49.72 | 420.03 | 0.12 | 248.52 | 0.906 | 0.02 | 155.53 | 405.70 | 0.38 | 249.32 | 0.701 | 0.04 | 140.23 | 406.59 | 0.34 | 249.26 | 0.730 | 0.04 |
| Days 1-14 vs Days 15-28 | -2.94 | 170.22 | -0.02 | 248.54 | 0.986 | 0.01 | 79.55 | 120.38 | 0.66 | 250.70 | 0.509 | 0.05 | 104.22 | 121.36 | 0.86 | 250.73 | 0.390 | 0.06 |
| Days 1-14 vs Days 15-28 * Condition | 224.18 | 244.71 | 0.92 | 249.69 | 0.360 | 0.06 |  |  |  |  |  |  |  |  |  |  |  |  |
| Day 15 vs all other days |  |  |  |  |  |  | 443.64 | 311.75 | 1.42 | 247.33 | 0.155 | 0.08 | 637.52 | 287.42 | 2.22 | 2160.88 | 0.027 | 0.09 |
| Day 15 vs all other days * Condition |  |  |  |  |  |  | -193.33 | 447.01 | -0.43 | 247.12 | 0.665 | 0.04 |  |  |  |  |  |  |
| Days 15-16 vs all other days |  |  |  |  |  |  |  |  |  |  |  |  | -301.30 | 278.73 | -1.08 | 419.97 | 0.280 | 0.07 |
| Days 15-16 vs all other days * Condition |  |  |  |  |  |  |  |  |  |  |  |  | -21.45 | 351.31 | -0.06 | 251.36 | 0.951 | 0.02 |

Note. ^1^ Condition was dummy-coded (0 = low saliency, 1 = high saliency).

Table S8. Fixed effects models testing the effects of saliency of researcher observation, controlled for gender, age, BMI, and tracker use duration (*N* = 235).

|  | Model 1 | | | | | | Model 2 | | | | | | Model 3 | | | | | |
| --- | --- | --- | --- | --- | --- | --- | --- | --- | --- | --- | --- | --- | --- | --- | --- | --- | --- | --- |
|  | b | SE | t | df | p | srs | b | SE | t | df | p | srs | b | SE | t | df | p | srs |
| Intercept | 8177.79 | 1021.69 | 8.00 | 229.47 | <0.001 |  | 8029.21 | 1011.34 | 7.94 | 228.37 | <0.001 |  | 8034.53 | 1011.52 | 7.94 | 228.37 | <0.001 |  |
| Condition^1^ | 152.52 | 434.02 | 0.35 | 225.38 | 0.725 | 0.04 | 315.06 | 413.29 | 0.76 | 225.89 | 0.446 | 0.06 | 296.51 | 413.87 | 0.72 | 226.30 | 0.474 | 0.06 |
| Gender: women vs men | 1700.45 | 474.47 | 3.58 | 226.01 | <0.001 | 0.12 | 1818.72 | 470.24 | 3.87 | 225.84 | <0.001 | 0.13 | 1802.64 | 470.32 | 3.83 | 225.83 | <0.001 | 0.13 |
| Gender: women vs gender neutral | -3732.91 | 3104.01 | -1.20 | 225.97 | 0.229 | 0.07 | -3117.46 | 3074.91 | -1.01 | 225.38 | 0.311 | 0.07 | -3242.77 | 3075.28 | -1.05 | 225.32 | 0.292 | 0.07 |
| Gender: women vs non-binary | 497.33 | 1582.97 | 0.31 | 225.21 | 0.753 | 0.04 | 622.00 | 1568.80 | 0.40 | 225.00 | 0.692 | 0.04 | 625.26 | 1569.06 | 0.40 | 224.98 | 0.690 | 0.04 |
| Gender: women vs would rather not say | -1029.49 | 2217.09 | -0.46 | 225.20 | 0.642 | 0.04 | -855.58 | 2197.23 | -0.39 | 224.99 | 0.697 | 0.04 | -876.54 | 2197.59 | -0.40 | 224.97 | 0.690 | 0.04 |
| Age | 66.30 | 17.14 | 3.87 | 225.67 | <0.001 | 0.13 | 64.11 | 16.99 | 3.77 | 225.60 | <0.001 | 0.13 | 64.46 | 16.99 | 3.79 | 225.57 | <0.001 | 0.13 |
| BMI | -102.04 | 34.81 | -2.93 | 225.73 | 0.003 | 0.11 | -101.26 | 34.50 | -2.93 | 225.63 | 0.003 | 0.11 | -100.87 | 34.51 | -2.92 | 225.61 | 0.003 | 0.11 |
| Tracker use duration^2^ | 31.08 | 451.27 | 0.07 | 226.00 | 0.945 | 0.02 | 147.84 | 447.28 | 0.33 | 225.88 | 0.741 | 0.04 | 130.27 | 447.35 | 0.29 | 225.86 | 0.771 | 0.04 |
| Days 1-14 vs Days 15-28 | -12.48 | 176.25 | -0.07 | 231.80 | 0.944 | 0.02 | 82.54 | 125.05 | 0.66 | 234.01 | 0.509 | 0.04 | 108.16 | 125.99 | 0.86 | 234.04 | 0.391 | 0.06 |
| Days 1-14 vs Days 15-28 * Condition | 248.35 | 253.12 | 0.98 | 232.99 | 0.327 | 0.06 |  |  |  |  |  |  |  |  |  |  |  |  |
| Day 15 vs all other days |  |  |  |  |  |  | 431.49 | 324.05 | 1.33 | 230.03 | 0.183 | 0.08 | 636.67 | 298.55 | 2.13 | 2612.74 | 0.033 | 0.10 |
| Day 15 vs all other days * Condition |  |  |  |  |  |  | -188.91 | 464.38 | -0.41 | 230.07 | 0.684 | 0.04 |  |  |  |  |  |  |
| Days 15-16 vs all other days |  |  |  |  |  |  |  |  |  |  |  |  | -293.28 | 294.88 | -0.99 | 368.47 | 0.320 | 0.06 |
| Days 15-16 vs all other days * Condition |  |  |  |  |  |  |  |  |  |  |  |  | -58.69 | 371.89 | -0.16 | 230.79 | 0.875 | 0.03 |

Note. ^1^ Condition was dummy-coded (0 = low saliency, 1 = high saliency). ^2^ Tracker use duration was dummy-coded for this analysis (0 = one year or less, 1 = more than a year).

Table S9. Fixed effects models for steps; only for participants who completed the questionnaires exactly 14 days apart (*N* = 110).

|  | Model 1 | | | | | | Model 2 | | | | | | Model 3 | | | | | |
| --- | --- | --- | --- | --- | --- | --- | --- | --- | --- | --- | --- | --- | --- | --- | --- | --- | --- | --- |
|  | b | SE | t | df | p | srs | b | SE | t | df | p | srs | b | SE | t | df | p | srs |
| Intercept | 7935.84 | 316.21 | 25.10 | 108.12 | <0.001 |  | 7936.79 | 316.30 | 25.09 | 108.10 | <0.001 |  | 7936.50 | 316.28 | 25.09 | 108.10 | <0.001 |  |
| Days 1-14 vs Days 15-28 | -15.91 | 170.89 | -0.09 | 108.45 | 0.926 | 0.03 | -27.96 | 170.62 | -0.16 | 109.18 | 0.870 | 0.04 | -20.08 | 173.88 | -0.12 | 108.64 | 0.908 | 0.03 |
| Day 15 vs all other days |  |  |  |  |  |  | 159.16 | 308.57 | 0.52 | 1474.94 | 0.606 | 0.07 | 250.80 | 418.61 | 0.60 | 1606.60 | 0.549 | 0.07 |
| Days 15-16 vs all other days |  |  |  |  |  |  |  |  |  |  |  |  | -99.43 | 322.79 | -0.31 | 221.98 | 0.758 | 0.05 |

Table S10. Fixed effects models for exploratory analyses of moderators; only for participants who completed the questionnaires exactly 14 days apart.

|  | Model 1 | | | | | | Model 2 | | | | | | Model 3 | | | | | |
| --- | --- | --- | --- | --- | --- | --- | --- | --- | --- | --- | --- | --- | --- | --- | --- | --- | --- | --- |
|  | b | SE | t | df | p | srs | b | SE | t | df | p | srs | b | SE | t | df | p | srs |
| ***Gender (N = 106)*** | | | | | | | | | | | | | | | | | | |
| Intercept | 7785.65 | 359.90 | 21.63 | 103.4 | <0.001 |  | 7653.44 | 357.96 | 21.38 | 106.04 | <0.001 |  | 7658.21 | 357.94 | 21.39 | 105.99 | <0.001 |  |
| Days 1-14 vs Days 15-28 | -292.70 | 192.33 | -1.52 | 103.0 | 0.128 | 0.12 | -62.35 | 174.46 | -0.36 | 105.09 | 0.721 | 0.06 | -53.32 | 177.54 | -0.30 | 104.65 | 0.764 | 0.05 |
| Gender^1^ | 973.26 | 759.55 | 1.28 | 104.6 | 0.200 | 0.11 | 1587.93 | 722.36 | 2.20 | 104.32 | 0.028 | 0.14 | 1564.54 | 723.46 | 2.16 | 104.36 | 0.031 | 0.14 |
| Days 1-14 vs Days 15-28 * Gender | 1067.34 | 411.24 | 2.60 | 104.0 | 0.009 | 0.16 |  |  |  |  |  |  |  |  |  |  |  |  |
| Day 15 vs all other days |  |  |  |  |  |  | -68.85 | 356.85 | -0.19 | 1658.98 | 0.847 | 0.04 | 145.17 | 425.13 | 0.34 | 1860.41 | 0.733 | 0.06 |
| Day 15 vs all other days * Gender |  |  |  |  |  |  | 482.83 | 742.14 | 0.65 | 1132.72 | 0.515 | 0.08 |  |  |  |  |  |  |
| Days 15-16 vs all other days |  |  |  |  |  |  |  |  |  |  |  |  | -228.63 | 358.31 | -0.64 | 230.39 | 0.523 | 0.08 |
| Days 15-16 vs all other days * Gender |  |  |  |  |  |  |  |  |  |  |  |  | 508.74 | 591.13 | 0.86 | 102.74 | 0.390 | 0.09 |
| ***SCO (N = 110*** | | | | | | | | | | | | | | | | | |  |
| Intercept | 7935.85 | 315.00 | 25.19 | 107.17 | <0.001 |  | 7937.35 | 315.01 | 25.20 | 107.29 | <0.001 |  | 7936.87 | 314.97 | 25.20 | 107.31 | <0.001 |  |
| Days 1-14 vs Days 15-28 | -15.67 | 171.60 | -0.09 | 107.54 | 0.927 | 0.03 | -28.44 | 170.56 | -0.17 | 109.21 | 0.868 | 0.04 | -20.45 | 173.86 | -0.12 | 108.66 | 0.906 | 0.03 |
| SCO | -64.97 | 47.81 | -1.36 | 106.81 | 0.174 | 0.11 | -68.96 | 45.82 | -1.51 | 107.66 | 0.132 | 0.12 | -66.29 | 45.83 | -1.45 | 107.65 | 0.148 | 0.11 |
| Days 1-14 vs Days 15-28 * SCO | -8.63 | 25.96 | -0.33 | 106.99 | 0.740 | 0.05 |  |  |  |  |  |  |  |  |  |  |  |  |
| Day 15 vs all other days |  |  |  |  |  |  | 160.07 | 308.71 | 0.52 | 1446.70 | 0.604 | 0.07 | 250.54 | 418.57 | 0.60 | 1612.82 | 0.550 | 0.07 |
| Day 15 vs all other days * SCO |  |  |  |  |  |  | -16.49 | 46.45 | -0.36 | 1050.75 | 0.723 | 0.06 |  |  |  |  |  |  |
| Days 15-16 vs all other days |  |  |  |  |  |  |  |  |  |  |  |  | -97.61 | 322.73 | -0.30 | 222.85 | 0.762 | 0.05 |
| Days 15-16 vs all other days * SCO |  |  |  |  |  |  |  |  |  |  |  |  | -34.13 | 36.68 | -0.93 | 106.74 | 0.352 | 0.09 |
| ***PA during the pre-study period (N = 109)*** | | | | | | | | | | | | | | | | | | |
| Intercept | 7880.97 | 78.11 | 100.89 | 2888.44 | <0.001 |  | 7881.50 | 78.14 | 100.86 | 2818.68 | <0.001 |  | 7881.44 | 77.78 | 101.33 | 2653.08 | <0.001 |  |
| Days 1-14 vs Days 15-28 | -17.49 | 180.06 | -0.10 | 152.46 | 0.923 | 0.03 | -30.26 | 184.10 | -0.16 | 119.09 | 0.869 | 0.04 | -22.49 | 187.27 | -0.12 | 118.64 | 0.904 | 0.03 |
| PA pre-study period | 0.99 | 0.02 | 41.91 | 2888.44 | <0.001 | 0.62 | 0.96 | 0.02 | 45.35 | 368.96 | <0.001 | 0.65 | 0.96 | 0.02 | 45.47 | 386.19 | <0.001 | 0.65 |
| Days 1-14 vs Days 15-28 * PA pre-study period | -0.15 | 0.05 | -2.78 | 151.31 | 0.005 | 0.16 |  |  |  |  |  |  |  |  |  |  |  |  |
| Day 15 vs all other days |  |  |  |  |  |  | 175.81 | 304.95 | 0.58 | 1580.81 | 0.564 | 0.07 | 263.74 | 413.84 | 0.64 | 1532.30 | 0.524 | 0.08 |
| Day 15 vs all other days * PA pre-study period |  |  |  |  |  |  | -0.07 | 0.09 | -0.76 | 1310.88 | 0.446 | 0.08 |  |  |  |  |  |  |
| Days 15-16 vs all other days |  |  |  |  |  |  |  |  |  |  |  |  | -97.34 | 324.06 | -0.30 | 193.08 | 0.764 | 0.05 |
| Days 15-16 vs all other days * PA pre-study period |  |  |  |  |  |  |  |  |  |  |  |  | -0.05 | 0.08 | -0.64 | 107.14 | 0.525 | 0.08 |

Note. Gender was dummy-coded for this analysis (0 = women, 1 = men).

Table S11. Fixed effects models testing the effects of saliency of researcher observation; only for participants who completed the questionnaires exactly 14 days apart (*N* = 110).

|  | Model 1 | | | | | | Model 2 | | | | | | Model 3 | | | | | |
| --- | --- | --- | --- | --- | --- | --- | --- | --- | --- | --- | --- | --- | --- | --- | --- | --- | --- | --- |
|  | b | SE | t | df | p | srs | b | SE | t | df | p | srs | b | SE | t | df | p | srs |
| Intercept | 7629.95 | 490.15 | 15.57 | 107.11 | <0.001 |  | 7608.57 | 478.13 | 15.91 | 112.10 | <0.001 |  | 7594.89 | 477.85 | 15.89 | 112.2 | <0.001 |  |
| Condition^1^ | 525.51 | 642.17 | 0.82 | 106.95 | 0.413 | 0.09 | 563.82 | 615.42 | 0.92 | 107.75 | 0.360 | 0.09 | 586.59 | 614.84 | 0.95 | 107.7 | 0.340 | 0.09 |
| Days 1-14 vs Days 15-28 | -46.58 | 266.24 | -0.17 | 106.64 | 0.861 | 0.04 | -27.73 | 170.61 | -0.16 | 109.12 | 0.871 | 0.04 | -19.70 | 173.91 | -0.11 | 108.6 | 0.910 | 0.03 |
| Days 1-14 vs Days 15-28 * Condition | 53.09 | 348.33 | 0.15 | 107.18 | 0.879 | 0.04 |  |  |  |  |  |  |  |  |  |  |  |  |
| Day 15 vs all other days |  |  |  |  |  |  | 329.19 | 477.82 | 0.69 | 1208.68 | 0.491 | 0.08 | 250.83 | 418.65 | 0.60 | 1600.0 | 0.549 | 0.07 |
| Day 15 vs all other days * Condition |  |  |  |  |  |  | -291.80 | 625.28 | -0.47 | 1061.31 | 0.641 | 0.07 |  |  |  |  |  |  |
| Days 15-16 vs all other days |  |  |  |  |  |  |  |  |  |  |  |  | 126.41 | 433.06 | 0.29 | 175.2 | 0.770 | 0.05 |
| Days 15-16 vs all other days * Condition |  |  |  |  |  |  |  |  |  |  |  |  | -387.59 | 494.51 | -0.78 | 106.8 | 0.433 | 0.08 |

Note. Condition was dummy-coded (0 = low saliency, 1 = high saliency).

Table S12. Fixed effects models for steps with covariates; only for participants who completed the questionnaires exactly 14 days apart, controlled for gender, age, BMI, and tracker use duration (*N* = 105).

|  | Model 1 | | | | | | Model 2 | | | | | | Model 3 | | | | | |
| --- | --- | --- | --- | --- | --- | --- | --- | --- | --- | --- | --- | --- | --- | --- | --- | --- | --- | --- |
|  | b | SE | t | df | p | srs | b | SE | t | df | p | srs | b | SE | t | df | p | srs |
| Intercept | 8853.41 | 1420.32 | 6.23 | 98.10 | <0.001 |  | 8849.75 | 1419.82 | 6.23 | 98.09 | <0.001 |  | 8854.40 | 1419.63 | 6.24 | 98.08 | <0.001 |  |
| Gender: women vs men | 1920.34 | 743.00 | 2.58 | 97.41 | 0.010 | 0.16 | 1933.28 | 742.69 | 2.60 | 97.37 | 0.009 | 0.16 | 1933.83 | 742.60 | 2.60 | 97.36 | 0.009 | 0.16 |
| Gender: women vs gender neutral | -3702.38 | 3076.40 | -1.20 | 97.19 | 0.229 | 0.11 | -3553.59 | 3074.81 | -1.16 | 97.11 | 0.248 | 0.11 | -3572.89 | 3074.38 | -1.16 | 97.09 | 0.245 | 0.11 |
| Gender: women vs non-binary | 214.90 | 2194.13 | 0.10 | 96.78 | 0.922 | 0.03 | 279.70 | 2193.26 | 0.13 | 96.75 | 0.899 | 0.04 | 290.02 | 2192.98 | 0.13 | 96.74 | 0.895 | 0.04 |
| Gender: women vs would rather not say | 702.12 | 3105.73 | 0.23 | 96.78 | 0.821 | 0.05 | 800.41 | 3104.49 | 0.26 | 96.75 | 0.797 | 0.05 | 783.55 | 3104.10 | 0.25 | 96.74 | 0.801 | 0.05 |
| Age | 60.96 | 25.97 | 2.35 | 97.00 | 0.019 | 0.15 | 60.53 | 25.96 | 2.33 | 96.98 | 0.020 | 0.15 | 60.58 | 25.95 | 2.33 | 96.97 | 0.020 | 0.15 |
| BMI | -116.93 | 47.23 | -2.48 | 96.99 | 0.013 | 0.15 | -117.40 | 47.21 | -2.49 | 96.96 | 0.013 | 0.15 | -117.57 | 47.20 | -2.49 | 96.95 | 0.013 | 0.15 |
| Tracker use duration^1^ | -135.75 | 662.25 | -0.20 | 97.12 | 0.838 | 0.04 | -100.52 | 662.00 | -0.15 | 97.10 | 0.879 | 0.04 | -103.83 | 661.92 | -0.16 | 97.09 | 0.875 | 0.04 |
| Days 1-14 vs Days 15-28 | -24.70 | 177.63 | -0.14 | 103.69 | 0.889 | 0.04 | -39.60 | 177.28 | -0.22 | 104.36 | 0.823 | 0.05 | -31.27 | 181.03 | -0.17 | 103.92 | 0.863 | 0.04 |
| Day 15 vs all other days |  |  |  |  |  |  | 196.97 | 320.20 | 0.62 | 1391.54 | 0.539 | 0.08 | 292.12 | 434.36 | 0.67 | 1480.15 | 0.501 | 0.08 |
| Days 15-16 vs all other days |  |  |  |  |  |  |  |  |  |  |  |  | -103.30 | 336.22 | -0.31 | 206.05 | 0.759 | 0.05 |

Note. ^1^ Tracker use duration was dummy-coded for this analysis (0 = one year or less, 1 = more than a year).

Table S13. Fixed effects models for exploratory analyses of moderators with covariates; only for participants who completed the questionnaires exactly 14 days apart, controlled for gender, age, BMI, and tracker use duration.

|  | Model 1 | | | | | | Model 2 | | | | | | Model 3 | | | | | |
| --- | --- | --- | --- | --- | --- | --- | --- | --- | --- | --- | --- | --- | --- | --- | --- | --- | --- | --- |
|  | b | SE | t | df | p | srs | b | SE | t | df | p | srs | b | SE | t | df | p | srs |
| ***Gender^1^ (N = 101)*** | | | | | | | | | | | | | | | | | | |
| Intercept | 9012.24 | 1409.40 | 6.39 | 97.29 | <0.001 |  | 8862.61 | 1409.66 | 6.29 | 97.06 | <0.001 |  | 8889.38 | 1409.26 | 6.31 | 97.04 | <0.001 |  |
| Age | 59.22 | 25.77 | 2.30 | 96.02 | 0.022 | 0.15 | 58.83 | 25.78 | 2.28 | 95.97 | 0.023 | 0.15 | 58.88 | 25.78 | 2.28 | 95.94 | 0.022 | 0.15 |
| BMI | -109.57 | 47.06 | -2.33 | 96.01 | 0.020 | 0-15 | -110.43 | 47.10 | -2.34 | 95.96 | 0.019 | 0.15 | -111.01 | 47.08 | -2.36 | 95.93 | 0.018 | 0.15 |
| Tracker use duration^2^ | -285.51 | 662.77 | -0.43 | 96.16 | 0.667 | 0.07 | -231.98 | 663.24 | -0.35 | 96.11 | 0.727 | 0.06 | -241.87 | 663.04 | -0.36 | 96.08 | 0.715 | .06 |
| Days 1-14 vs Days 15-28 | -314.20 | 199.97 | -1.57 | 98.05 | 0.116 | 0.12 | -73.97 | 181.63 | -0.41 | 100.13 | 0.684 | 0.06 | -64.43 | 185.19 | -0.35 | 99.77 | 0.728 | 0.06 |
| Gender^3^ | 1157.64 | 782.83 | 1.48 | 98.96 | 0.139 | 0.12 | 1838.92 | 740.38 | 2.48 | 96.53 | 0.013 | 0.16 | 1805.23 | 742.33 | 2.43 | 96.69 | 0.015 | 0.16 |
| Days 1-14 vs Days 15-28 * Gender | 1119.11 | 426.67 | 2.62 | 99.01 | 0.009 | 0.16 |  |  |  |  |  |  |  |  |  |  |  |  |
| Day vs all other days |  |  |  |  |  |  | -20.07 | 371.41 | -0.05 | 1508.54 | 0.957 | 0.02 | 184.22 | 441.90 | 0.42 | 1670.66 | 0.677 | 0.06 |
| Day 15 vs all other days * Gender |  |  |  |  |  |  | 418.67 | 770.03 | 0.54 | 1049.3 | 0.587 | 0.07 |  |  |  |  |  |  |
| Days 15-16 vs all other days |  |  |  |  |  |  |  |  |  |  |  |  | -230.33 | 374.60 | -0.61 | 212.03 | 0.539 | 0.08 |
| Days 15-16 vs all other days * Gender |  |  |  |  |  |  |  |  |  |  |  |  | 491.67 | 616.63 | 0.80 | 97.70 | 0.425 | 0.09 |
| ***SCO (N = 105)*** | | | | | | | | | | | | | | | | | | |
| Intercept | 8962.11 | 1434.30 | 6.25 | 97.11 | <0.001 |  | 8963.57 | 1433.51 | 6.25 | 97.09 | <0.001 |  | 8972.81 | 1433.27 | 6.26 | 97.07 | <0.001 |  |
| Gender: women vs men | 1827.35 | 758.52 | 2.41 | 96.42 | 0.016 | 0.15 | 1838.52 | 758.06 | 2.43 | 96.37 | 0.015 | 0.15 | 1838.23 | 757.94 | 2.43 | 96.35 | 0.015 | 0.15 |
| Gender: women vs gender neutral | -3758.60 | 3086.68 | -1.22 | 96.19 | 0.223 | 0.11 | -3580.49 | 3084.49 | -1.16 | 96.09 | 0.246 | 0.11 | -3605.92 | 3083.90 | -1.17 | 96.06 | 0.242 | 0.11 |
| Gender: women vs non-binary | 24.07 | 2219.97 | 0.01 | 95.80 | 0.991 | 0.01 | 93.91 | 2218.70 | 0.04 | 95.76 | 0.966 | 0.02 | 111.98 | 2218.33 | 0.05 | 95.74 | 0.960 | 0.02 |
| Gender: women vs would rather not say | 1040.40 | 3158.52 | 0.33 | 95.79 | 0.742 | 0.06 | 1176.12 | 3156.70 | 0.37 | 95.75 | 0.709 | 0.06 | 1151.98 | 3156.17 | 0.36 | 95.73 | 0.715 | 0.06 |
| Age | 53.71 | 28.32 | 1.90 | 96.11 | 0.058 | 0.13 | 52.85 | 28.30 | 1.87 | 96.09 | 0.062 | 0.13 | 52.86 | 28.30 | 1.87 | 96.07 | 0.062 | 0.13 |
| BMI | -113.62 | 47.64 | -2.38 | 96.00 | 0.017 | 0.15 | -114.05 | 47.61 | -2.40 | 95.97 | 0.017 | 0.15 | -114.33 | 47.60 | -2.40 | 95.95 | 0.016 | 0.15 |
| Tracker use duration^2^ | -67.11 | 672.53 | -0.10 | 96.11 | 0.921 | 0.03 | -21.06 | 672.15 | -0.03 | 96.08 | 0.975 | 0.02 | -25.20 | 672.04 | -0.04 | 96.06 | 0.970 | 0.02 |
| Days 1-14 vs Days 15-28 | -24.36 | 178.46 | -0.14 | 102.72 | 0.891 | 0.04 | -39.67 | 177.25 | -0.22 | 104.34 | 0.823 | 0.05 | -31.20 | 181.02 | -0.17 | 103.89 | 0.863 | 0.04 |
| SCO | -29.91 | 54.28 | -0.55 | 99.49 | 0.582 | 0.07 | -33.54 | 51.57 | -0.65 | 96.23 | 0.516 | 0.08 | -30.87 | 51.66 | -0.60 | 96.43 | 0.550 | 0.08 |
| Days 1-14 vs Days 15-28 * SCO | -5.66 | 27.14 | -0.21 | 102.22 | 0.835 | 0.04 |  |  |  |  |  |  |  |  |  |  |  |  |
| Day 15 vs all other days |  |  |  |  |  |  | 197.52 | 320.35 | 0.62 | 1362.34 | 0.538 | 0.08 | 292.25 | 434.29 | 0.67 | 1501.37 | 0.501 | 0.08 |
| Day 15 vs all other days * SCO |  |  |  |  |  |  | -19.51 | 48.46 | -0.40 | 1003.93 | 0.687 | 0.06 |  |  |  |  |  |  |
| Days 15-16 vs all other days |  |  |  |  |  |  |  |  |  |  |  |  | -102.68 | 338.13 | -0.30 | 257.94 | 0.761 | 0.05 |
| Days 15-16 vs all other days * SCO |  |  |  |  |  |  |  |  |  |  |  |  | -34.10 | 38.46 | -0.89 | 101.11 | 0.375 | 0.09 |
| ***PA during the pre-study period (N = 104)*** | | | | | | | | | | | | | | | | | | |
| Intercept | 7819.89 | 350.10 | 22.34 | 351.35 | <0.001 |  | 7818.02 | 349.38 | 22.38 | 362.55 | <0.001 |  | 7826.35 | 347.58 | 22.52 | 345.44 | <0.001 |  |
| Gender: women vs men | 299.55 | 185.36 | 1.62 | 319.68 | 0.106 | 0.12 | 289.42 | 184.93 | 1.57 | 328.23 | 0.118 | 0.12 | 289.05 | 183.97 | 1.57 | 312.68 | 0.116 | 0.12 |
| Gender: women vs gender neutral | -15.32 | 745.04 | -0.02 | 324.80 | 0.984 | 0.01 | -62.14 | 743.33 | -0.08 | 334.00 | 0.933 | 0.03 | -72.59 | 739.38 | -0.10 | 319.14 | 0.922 | 0.03 |
| Gender: women vs non-binary | 163.09 | 527.42 | 0.31 | 315.02 | 0.757 | 0.05 | 136.83 | 526.14 | 0.26 | 323.52 | 0.795 | 0.05 | 154.18 | 523.41 | 0.29 | 307.76 | 0.768 | ß.05 |
| Gender: women vs would rather not say | 569.15 | 746.83 | 0.76 | 315.02 | 0.446 | 0.09 | 528.17 | 745.02 | 0.71 | 323.53 | 0.478 | 0.08 | 510.51 | 741.15 | 0.69 | 307.76 | 0.491 | 0.08 |
| Age | 4.28 | 6.49 | 0.66 | 317.74 | 0.510 | 0.08 | 4.47 | 6.47 | 0.69 | 325.99 | 0.490 | 0.08 | 4.45 | 6.44 | 0.69 | 310.09 | 0.490 | 0.08 |
| BMI | -4.35 | 11.84 | -0.37 | 317.84 | 0.714 | 0.06 | -3.85 | 11.82 | -0.33 | 326.16 | 0.744 | 0.06 | -4.13 | 11.76 | -0.35 | 310.30 | 0.725 | 0.06 |
| Tracker use duration^2^ | -42.97 | 162.74 | -0.26 | 318.97 | 0.792 | 0.05 | -57.95 | 162.37 | -0.36 | 327.23 | 0.721 | 0.06 | -59.22 | 161.54 | -0.37 | 311.30108.38 | 0.714 | 0.06 |
| Days 1-14 vs Days 15-28 | -11.34 | 185.41 | -0.06 | 113.04 | 0.951 | 0.02 | -38.85 | 189.92 | -0.20 | 112.87 | 0.838 | 0.04 | -30.62 | 193.57 | -0.16 | 108.38 | 0.874 | 0.04 |
| PA pre-study period | 0.98 | 0.03 | 38.56 | 2809.79 | <0.001 | 0.61 | 0.95 | 0.02 | 41.62 | 328.39 | <0.001 | 0.63 | 0.95 | 0.02 | 41.74 | 304.9 | <0.001 | 0.63 |
| Days 1-14 vs Days 15-28 * PA pre-study period | -0.16 | 0.06 | -2.84 | 112.13 | 0.005 | 0.17 |  |  |  |  |  |  |  |  |  |  |  |  |
| Day 15 vs all other days |  |  |  |  |  |  | 222.01 | 317.05 | 0.70 | 1517.51 | 0.484 | 0.08 | 308.29 | 430.53 | 0.72 | 1347.92 | 0.474 | 0.08 |
| Day 15 vs all other days * PA pre-study period |  |  |  |  |  |  | -0.08 | 0.10 | -0.86 | 1228.19 | 0.389 | 0.09 |  |  |  |  |  |  |
| Days 15-16 vs all other days |  |  |  |  |  |  |  |  |  |  |  |  | -98.38 | 338.35 | -0.29 | 179.44 | 0.771 | 0.05 |
| Days 15-16 vs all other days * PA pre-study period |  |  |  |  |  |  |  |  |  |  |  |  | -0.06 | 0.08 | -0.72 | 101.54 | 0.469 | 0.08 |

Note. ^1^ Since gender was introduced as a moderator, it was not introduced as a covariate in this model. ^2^ Tracker use duration was dummy-coded for this analysis (0 = one year or less, 1 = more than a year). ^3^ Gender was dummy-coded for this analysis (0 = women, 1 = men).

Table S14. Fixed effects models testing the effects of saliency of researcher observation with covariates; only for participants who completed the questionnaires exactly 14 days apart, controlled for gender, age, BMI, and tracker use duration (*N* = 105).

|  | Model 1 | | | | | | Model 2 | | | | | | Model 3 | | | | | |
| --- | --- | --- | --- | --- | --- | --- | --- | --- | --- | --- | --- | --- | --- | --- | --- | --- | --- | --- |
|  | b | SE | t | df | p | srs | b | SE | t | df | p | srs | b | SE | t | df | p | srs |
| Intercept | 8612.44 | 1445.94 | 5.96 | 98.53 | <0.001 |  | 8596.26 | 1439.09 | 5.97 | 97.11 | <0.001 |  | 8576.66 | 1438.88 | 5.96 | 97.14 | <0.001 |  |
| Condition^1^ | 631.48 | 670.23 | 0.94 | 96.95 | 0.346 | 0.09 | 651.32 | 629.52 | 1.03 | 96.09 | 0.301 | 0.10 | 681.58 | 630.39 | 1.08 | 96.20 | 0.280 | 0.10 |
| Gender: women vs men | 1988.13 | 745.33 | 2.67 | 96.35 | 0.008 | 0.16 | 2001.05 | 744.96 | 2.69 | 96.32 | 0.007 | 0.16 | 2000.98 | 744.77 | 2.69 | 96.09 | 0.007 | 0.16 |
| Gender: women vs gender neutral | -3286.00 | 3103.63 | -1.06 | 96.17 | 0.290 | 0.10 | -3142.00 | 3101.89 | -1.01 | 96.11 | 0.311 | 0.10 | -3151.05 | 3101.10 | -1.02 | 96.31 | 0.310 | 0.10 |
| Gender: women vs non-binary | 632.33 | 2232.22 | 0.28 | 95.76 | 0.777 | 0.05 | 694.04 | 2231.22 | 0.31 | 95.74 | 0.756 | 0.05 | 690.80 | 2230.65 | 0.31 | 95.73 | 0.757 | 0.05 |
| Gender: women vs would rather not say | 1095.78 | 3127.46 | 0.35 | 95.76 | 0.726 | 0.06 | 1192.01 | 3126.06 | 0.38 | 95.74 | 0.703 | 0.06 | 1189.47 | 3125.25 | 0.38 | 95.73 | 0.704 | 0.06 |
| Age | 62.02 | 25.98 | 2.39 | 95.98 | 0.017 | 0.15 | 61.60 | 25.97 | 2.37 | 95.96 | 0.018 | 0.15 | 61.66 | 25.97 | 2.37 | 95.95 | 0.018 | 0.15 |
| BMI | -123.22 | 47.65 | -2.59 | 95.95 | 0.010 | 0.16 | -123.67 | 47.63 | -2.60 | 95.93 | 0.009 | 0.16 | -123.63 | 47.62 | -2.60 | 95.92 | 0.009 | 0.16 |
| Tracker use duration^2^ | -178.37 | 663.61 | -0.27 | 96.13 | 0.788 | 0.05 | -142.91 | 663.32 | -0.22 | 96.12 | 0.829 | 0.05 | -144.08 | 663.16 | -0.22 | 96.11 | 0.828 | 0.05 |
| Days 1-14 vs Days 15-28 | -21.77 | 276.61 | -0.08 | 101.83 | 0.937 | 0.03 | -39.40 | 177.27 | -0.22 | 104.31 | 0.824 | 0.05 | -30.87 | 181.04 | -0.17 | 103.88 | 0.865 | 0.04 |
| Days 1-14 vs Days 15-28 * Condition | -4.50 | 362.09 | -0.01 | 102.42 | 0.990 | 0.01 |  |  |  |  |  |  |  |  |  |  |  |  |
| Day 15 vs all other days |  |  |  |  |  |  | 408.50 | 495.38 | 0.82 | 1150.59 | 0.410 | 0.09 | 292.26 | 434.41 | 0.67 | 1470.42 | 0.501 | 0.08 |
| Day 15 vs all other days * Condition |  |  |  |  |  |  | -363.43 | 648.74 | -0.56 | 1014.50 | 0.575 | 0.07 |  |  |  |  |  |  |
| Days 15-16 vs all other days |  |  |  |  |  |  |  |  |  |  |  |  | 184.58 | 450.59 | 0.41 | 166.44 | 0.682 | 0.06 |
| Days 15-16 vs all other days * Condition |  |  |  |  |  |  |  |  |  |  |  |  | -494.80 | 515.04 | -0.96 | 101.83 | 0.337 | 0.10 |

Note. ^1^ Condition was dummy-coded (0 = low saliency, 1 = high saliency). ^2^ Tracker use duration was dummy-coded for this analysis (0 = one year or less, 1 = more than a year).
